# NFKB2 mediates colorectal cancer cell immune escape and metastasis in a STAT2/PD‐L1‐dependent manner

**DOI:** 10.1002/mco2.521

**Published:** 2024-04-24

**Authors:** Jiwei Zhang, Fen Ma, Zhe Li, Yuan Li, Xun Sun, Mingxu Song, Fan Yang, Enjiang Wu, Xiaohui Wei, Zhengtao Wang, Li Yang

**Affiliations:** ^1^ Shanghai Key Laboratory of Compound Chinese Medicines The MOE Key Laboratory for Standardization of Chinese Medicines Institute of Chinese Materia Medica Shanghai University of Traditional Chinese Medicine Shanghai China; ^2^ Academy of Integrative Medicine Shanghai University of Traditional Chinese Medicine Shanghai China; ^3^ Gastrointestinal Surgery Longhua Hospital Shanghai University of Traditional Chinese Medicine Shanghai China; ^4^ Human Reproductive and Genetic Center Affiliated Hospital of Jiangnan University Jiangsu China

**Keywords:** colorectal cancer, immunotherapy, nuclear factor kappa B subunit 2, programmed death ligand 1, signal transducer and activator of transcription 2

## Abstract

This study systematically analyzed the molecular mechanism and function of nuclear factor kappa B subunit 2 (NFKB2) in colorectal cancer (CRC) to investigate the potential of NFKB2 as a therapeutic target for CRC. Various experimental techniques, including RNA sequencing, proteome chip assays, and small molecule analysis, were used to obtain a deeper understanding of the regulation of NFKB2 in CRC. The results revealed that NFKB2 was upregulated in a significant proportion of patients with advanced hepatic metastasis of CRC. NFKB2 played an important role in promoting tumor growth through CD8^+^ T‐cell exhaustion. Moreover, NFKB2 directly interacted with signal transducer and activator of transcription 2 (STAT2), leading to increased phosphorylation of STAT2 and the upregulation of programmed death ligand 1 (PD‐L1). Applying a small molecule inhibitor of NFKB2 (Rg5) led to a reduction in PD‐L1 expression and improved response to programmed death‐1 blockade‐based immunotherapy. In conclusion, the facilitated NFKB2‐STAT2/PD‐L1 axis may suppress immune surveillance in CRC and targeting NFKB2 may enhance the efficacy of immunotherapeutic strategies. Our results provide novel insights into the molecular mechanisms underlying the contribution of NFKB2 in CRC immune escape.

## Graphical abstract

Amplification of 10q24.32 in CRC resulted in upregulation of NFKB2 protein levels, which directly interacts with STAT2, leading to STAT2 protein stabilization and phosphorylation, and consequently, further upregulating PD‐L1. Rg5, a small‐molecule inhibitor of NFKB2, could break the NFKB2‐STAT2 interaction, with significant reduced PD‐L1 expression and alleviated CD8^+^ T‐cell exhaustion, leading to an enhanced sensitivity to PD‐1 blockade‐based immunotherapy.

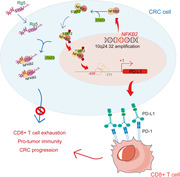

## 1 INTRODUCTION

More than 1.8 million new colorectal cancer (CRC) cases and approximately ∼0.8 million CRC‐related deaths are reported annually,^1^, ^2^ making it the second leading cause of cancer‐related mortality worldwide.^3^ Most CRC patients eventually develop distant metastasis within 5 years of the primary tumor diagnosis (metachronous metastasis patients), suggesting metastasis as a potential antitumor therapeutic target for improving survival rates in CRC patients. At the site of distant CRC metastasis, the accumulation of innate immune cells and the presence of a tumor‐conducive inflammatory environment results in immune dysregulation preceding the arrival of metastatic cells. Inflammatory factors play an indispensable role in the initiation and progression of the metastatic cascade and metastasis. Changes in the inflammatory factor expression in the tumor microenvironment promote tumor diffusion, colonization, and metastasis in the liver.^4^ Moreover, several cytokines and other molecules secreted by CRC cells have specific functions in inflammation, immunity, and CRC development.

Recent studies have suggested that the nuclear factor κB (NF‐κB) signal cascade mediates gastrointestinal inflammation in immune‐related disorders such as inflammatory bowel disease and malignant CRC.^5^, ^6^ NF‐κB, a key regulator of inflammation, is the link between inflammation and cancer. An immune response is associated with colon cancer treatment that might be due to NF‐κB activation contributing to immune activation and cancer cell survival.^7^, ^8^ Substantial evidence implicates NF‐κB in all stages of CRC development, from early adenoma to invasive cancer and metastasis.^9^ Among the NF‐κB family of transcription factors, NFKB2 (p100/p52) is proteolytically cleaved in the proteasome to produce p52, which then binds Rel proteins to form heterodimeric complexes that bind DNA and regulate the transcription of genes necessary for inflammatory and immune responses.^10^, ^11^ Elevated expression levels of NFKB2 and the activation of the alternative pathway of NF‐κB have been confirmed in non‐small cell lung cancer, breast cancer, prostate tumors, and several cancer cell lines.^12^, ^13^, ^14^, ^15^ The activation of NF‐κB pathways has been associated with unfavorable prognosis, indicating components within these pathways as potential prognostic markers or therapeutic targets for malignancies.^16^

In the context of CRC, NFKB2 has emerged as a significant player. Notably, Moy et al. uncovered a clinically relevant pathway involving ITPR3 and RELB that drives CRC cell survival and liver metastasis.^17^ Additionally, Tao et al. elucidated a non‐canonical NF‐κB signaling pathway where upregulated Bcl‐3 contributes to the development of colorectal tumors.^18^ Despite these advances, the impact of NFKB2 on the immune microenvironment and its interaction with other immune cells in CRC remain largely unexplored. Our study found that NFKB2 is overexpressed in CRC and CRC‐hepatic metastasis samples, promoting tumor formation and metastasis in CRC models. NFKB2 directly binds to and stabilizes signal transducer and activator of transcription 2 (STAT2), leading to increased programmed death ligand 1 (PD‐L1) expression and activity while reducing CD8^+^ T‐cell numbers and IFN‐γ secretion. We identified ginsenoside Rg5 as an inhibitor of NFKB2 activity, which resulted in increased CD8^+^ T‐cell populations, reduced tumor size, and inhibited PD‐L1 expression. These findings shed light on the role of NFKB2 in CRC immune escape. By shedding light on the role of NFKB2 on the CRC microenvironment, our research offers valuable insights that can contribute to a more comprehensive understanding of CRC pathogenesis and provide novel therapeutic strategies. We believe that our study presents a fresh perspective on the unique mechanisms of NFKB2 in mediating immune escape in the CRC microenvironment, demonstrating its novelty and potential clinical significance.

## 2 RESULTS

### 2.1 NFKB2 expression is significantly elevated in advanced metastatic CRC and is positively correlated with patient survival

**FIGURE 1:**
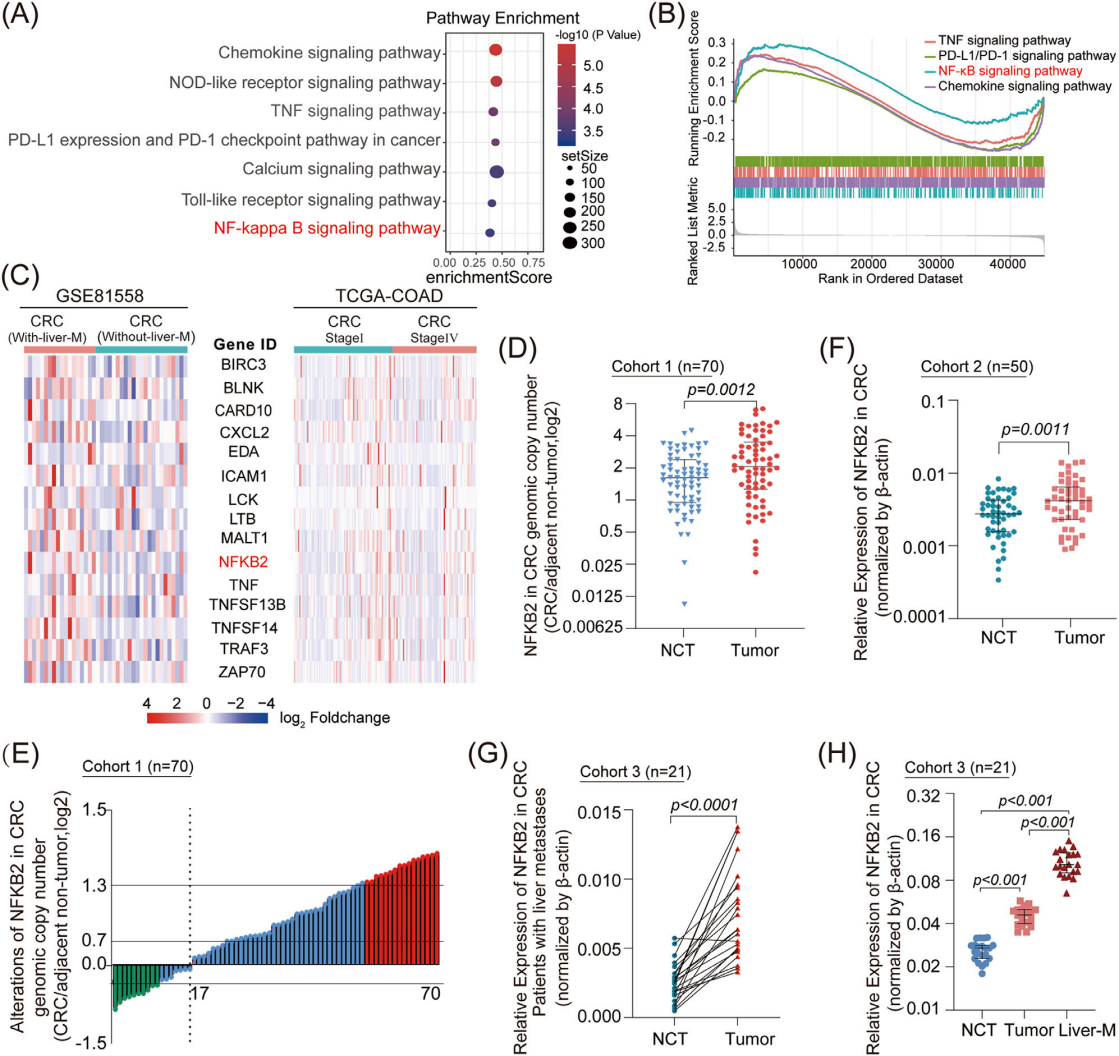
Expression of nuclear factor kappa B subunit 2 (NFKB2) is significantly elevated in advanced metastatic colorectal cancer (CRC) and is positively correlated with patient prognosis. (A) Gene Ontology pathway enrichment analysis based on the differentially expressed genes (DEGs) pattern of GSE81558 samples. (B) Functional annotations were conducted on the samples from GSE81558, unveiling the most prominently ranked signaling pathways. (C) The significant difference in the expression levels of nuclear factor κB (NF‐κB)‐related genes between the samples with and without hepatic metastasis in GSE81558 cohort, or between the samples in stages I and IV in the TCGA‐COAD cohort. (D and E) Genomic copy number analysis for NFKB2 in paired CRC tissues and the corresponding non‐cancerous tissues (NCTs) in Cohort 1. (F) The mRNA levels of NFKB2 in paired CRC tissues and the corresponding NCTs in Cohort 2. (G) The mRNA levels of NFKB2 in paired CRC samples and the corresponding NCTs from the patients with hepatic metastasis in Cohort 3. (H) The mRNA levels of NFKB2 in the grouped CRC tissues, the corresponding NCTs, and the corresponding hepatic metastatic loci form the same patient in Cohort 3. Values are expressed as median with interquartile range (D, F, and H). β‐Actin was used as the internal control (F–H). Values are expressed as mean ± SEMs.

We first performed differential gene expression analysis using the 23 primary colorectal tumor samples and 19 colorectal hepatic metastasis samples from the GSE81558 database to identify genes enriched in CRC patients with hepatic metastasis (Figure S1A). These metastasis‐upregulated genes were significantly elevated in the tumor necrosis factor (TNF) and NF‐κB signaling pathways (Figure 1A). GSEA results revealed that the NF‐κB signaling pathway had the highest enrichment score (Figure 1B). The activation of the NF‐κB signaling pathway was also found in the 75 Stage I samples and 51 Stage IV samples of patients in the TCGA‐COAD database (Figure S1B,C), suggesting that the abnormal upregulation of the NF‐κB signaling pathway is closely correlated with advanced CRC. Further exploration of the expression levels of NF‐κB signaling‐associated genes demonstrated that NFKB2 was significantly upregulated in metastatic CRCs (Figure 1C). Based on the analysis of a small genetic sample of 10 paired CRC/adjacent non‐tumor samples, NFKB2 was among the top‐ranked deregulated genes and its expression was significantly upregulated (Figure S1D). NFKB2 showed significantly higher genomic copy numbers in CRC samples compared to those in the nontumor samples (Figure 1D,E), and the mRNA and proteins levels of NFKB2 were also upregulated in CRC samples (Figure 1F and Figure S2A). Moreover, high NFKB2 mRNA levels were correlated with bigger tumor size, poorer differentiation grade, and worse tumor node metastasis (TNM) stage (Table 1 & Table S1). Importantly, distinctly upregulated NFKB2 mRNA levels were observed in CRC samples with advanced hepatic metastases (Figure 1G). Furthermore, NFKB2 showed the highest mRNA levels in the CRC specimen in the hepatic metastasis loci, which was higher than that in the CRC specimen in situ and the non‐tumor tissue from the same patient (Figure 1H), suggesting that NFKB2 may be one of the main factors driving CRC metastasis, especially CRC‐hepatic metastasis. In particular, high NFKB2 expression levels remarkably correlated with poor clinical outcomes in CRC patients in the TCGA‐COAD cohort (Figure S2B).

**TABLE 1:** The relationship between nuclear factor kappa B subunit 2 (NFKB2) expression and clinicopathological features of patients with colorectal cancer.

Characteristics	No. of patients	NFKB2 (low)	NFKB2 (High)	*χ2*	*P*‐value
Sex				0.8749	0.3496
male	39	22	17		
female	31	14	17		
Age (year)				1.3731	0.2414
< 60	42	24	18		
≥ 60	28	12	16		
Tumor size (cm)				6.8821	***0.0087***
< 5	40	26	14		
≥ 5	30	10	20		
Differentiation grade				11.7210	***0.0006***
Well and moderately	52	33	19		
Poorly	18	3	15		
Tumor stage				13.1110	***0.0003***
I‐II	32	24	8		
III‐IV	38	12	26		

*Note*: Pearson's chi‐squared test. Bold and italic indicates significance. Low, mRNA levels in T tissues ≤ median value; High, mRNA levels in T tissues > median value.

### 2.2 NFKB2 promotes CRC tumor formation in multiple mouse tumor models

**FIGURE 2:**
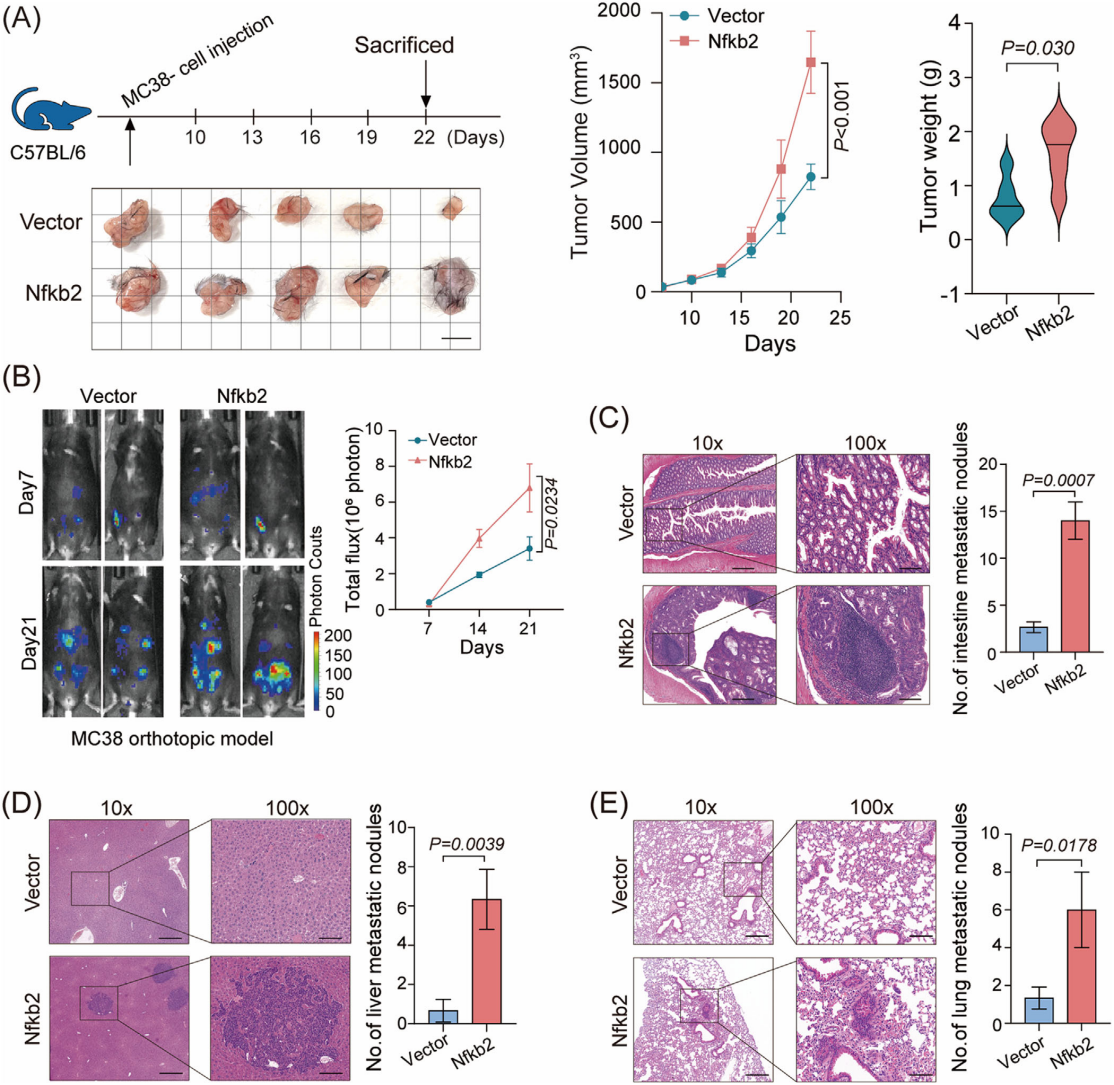
Nuclear factor kappa B subunit 2 (NFKB2) promotes colorectal cancer (CRC) tumor formation in multiple mouse tumor models. **(A)** The tumor volumes and tumor weights of the Nfkb2‐MC38‐luciferase and vector‐MC38‐luciferase groups in C57BL/6 mouse model. The standard and grid lines were 1 cm long. **(B)** Following in situ inoculation of Nfkb2‐MC38‐luciferase and vector‐MC38‐luciferase in the C57BL/6 mouse intestines, the bioluminescent intensity of mice was measured at different time points. Representative bioluminescent images on days 7 and 21 are shown on the left, accompanied by the corresponding statistical results on the right. **(C)** Statistical analysis of intestinal metastasis loci of the Nfkb2‐MC38 and vector‐MC38 groups in C57BL/6 mouse model, 9 weeks after the injection of the indicated cells. **(D)** Statistical analysis of liver metastases of the Nfkb2‐MC38 and vector‐MC38 groups in C57BL/6 mouse model, 9 weeks after the injection of the indicated cells. **(E)** Statistical analysis of lung metastases of the Nfkb2‐MC38 and vector‐MC38 groups in C57BL/6 mouse model, 9 weeks after the injection of the indicated cells. Values are expressed as mean ± SEMs, *n* = 5 in (**A and B**) and *n* = 3 in (**C–E**). Original magnification = 200× (**C–E**).

Next, we further investigated the biological functions of NFKB2 in CRC cell line. The results indicate that the highly expressed NFKB2 in the NFKB2‐overexpressing cell lines only exerted a subtle effect on the proliferation of HT29 cells, with an approximate 10% enhancement in proliferation observed on the seventh day (Figure S3A). Consequently, NFKB2 overexpression should have shown its ability on the tumorigenesis of HT‐29 cells in vivo, but surprisingly, there was no obvious effects that NFKB2 promote the tumor growth of HT‐29 xenografts in nude mouse models (Figure S3B). Considering the role of NFKB2 in the immune response,^19^ we further examined the effects of NFKB2 overexpression on CRC cells using syngeneic mouse models. The results revealed that Nfkb2 significantly promoted subcutaneous tumor formation in MC38‐C57BL/6 mouse models and CT26‐BALB/c mouse models (Figure 2A and Figure S3C). We also evaluated the promoting effects of NFKB2 on cell metastasis by establishing an orthotropic transplantation model and found that NFKB2 significantly promote hepatic metastasis in MC38 and CT26 CRC tumor models (Figure 2B and Figure S3D). On hematoxylin‐eosin staining the number of metastatic foci derived from NFKB2 cells dramatically increased in the intestine, liver, and lung sections (Figure 2C–E). Taken together, these findings suggest that NFKB2 acts as an oncogenic driver in CRC development and progression.

### 2.3 NFKB2 participates in the NF‐κB inhibitory signaling pathway and programmed death‐1/PD‐L1 blockade in CRC cells

**FIGURE 3:**
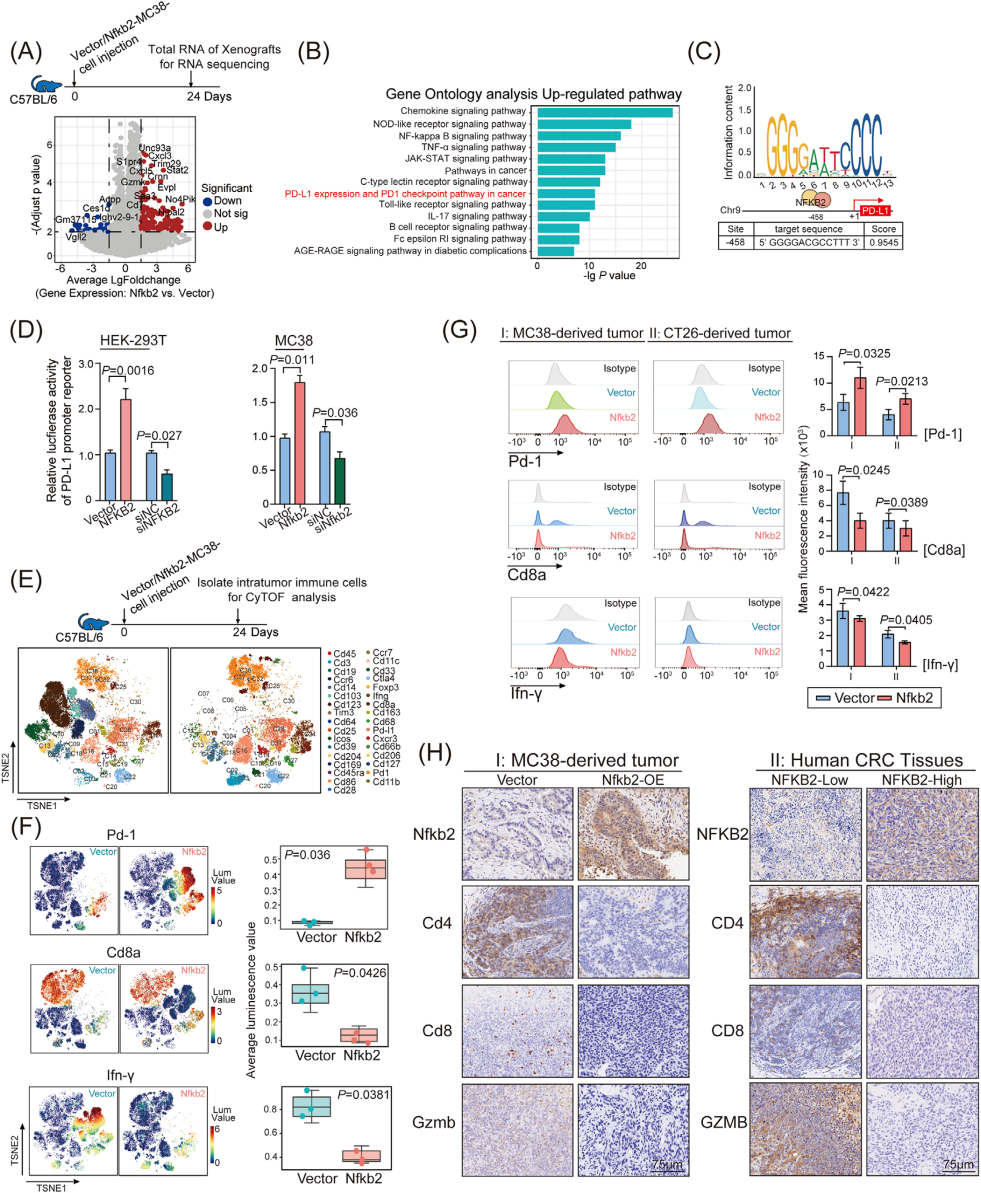
Nuclear factor kappa B subunit 2 (NFKB2) promotes the development of colorectal cancer (CRC) by promoting CD8^+^ T‐cell exhaustion through programmed death ligand 1 (PD‐L1). **(A)** Volcano map of the differentially expressed gene (DEG) pattern of the subcutaneous xenografts derived from Nfkb2‐MC38 cells compared to vector‐MC38 cells in C57BL/6 mouse model. **(B)** Gene Ontology (GO) pathway enrichment analysis based on the DEG pattern of the subcutaneous xenografts derived from Nfkb2‐MC38 cells compared to vector‐MC38 cells in C57BL/6 mouse model. **(C)** The predicted NFKB2 binding site in the PD‐L1 promoter region (the JASPR database). **(D)** The promoting effect of NFKB2 knockdown or overexpression in HEK‐293T and MC38 on the luciferase activity of the PD‐L1 promoter reporter. **(E)** The t‐distributed stochastic neighbor embedding (t‐SNE) map derived from mass cytometry by time‐of‐flight (CyTOF) analysis of intrahepatic immune cells obtained from the subcutaneous xenografts of Nfkb2‐MC38 or vector‐MC38 groups in C57BL/6 mouse model. The cells were colored according to clusters identified by Rphenograph. Clusters were grouped by expression profile. **(F)** CyTOF results showed the effects of NFKB2 on the expression levels of Pd‐1, Cd8a, and IFN‐γ in these intrahepatic immune cells. **(G)** The expression levels of Pd‐1, Cd8a, and IFN‐γ in the intrahepatic Cd45^+^ cells obtained from the subcutaneous xenografts of Nfkb2‐MC38 or vector‐MC38 groups in C57BL/6 mouse model. The expression levels were examined by flow cytometry (FACS) and presented as mean fluorescence intensity (MFI). **(H)** Left: The expression levels of Nfkb2, Cd4, Cd8, and Gzmb were evaluated by immunohistochemistry in the tumor tissues derived from C57BL/6 mice that were subcutaneously transplanted with Nfkb2‐MC38 or vector‐MC38 cells. Right: The expression levels of NFKB2, CD4, CD8, and GZMB in the tumor tissues of CRC patients stratified into the NFKB2‐high and NFKB2‐low groups were assessed using immunohistochemistry. Values are expressed as mean ± SEMs, *n* = 3 in (D, F, and G).

NFKB2, a key regulator of intracellular TNFα signaling, has long been reported to be closely associated with immune‐regulation,^20^, ^21^, ^22^ which had no effect on HT‐29 cell growth in our nude mouse models. As crosstalk between tumor cells and the immune system is generally considered a pivotal factor in CRC development,^23^ we hypothesized that the murine immune system may contribute to the tumor‐promoting function of NFKB2 in syngeneic mouse models. To explore the mechanisms underlying the tumor proliferative effect of NFKB2 in syngeneic mouse models, we analyzed the transcriptomic profiles of MC38 tumors in C57BL/6 mouse model by RNA‐seq assays (Figure S4), and an obvious difference existed between the transcriptomic profiles of xenografts with or without Nfkb2 overexpression (Figure 3A). Kyoto Encyclopedia of Genes and Genomes (KEGG) pathway enrichment analysis and hierarchical clustering heatmap analysis showed that the NF‐κB pathway, PD‐L1 expression, programmed death‐1 (PD‐1) checkpoint pathway, and JAK‐STAT pathway were the top‐ranked signaling pathways responding to Nfkb2 overexpression (Figure 3B). As previously reported, the mRNA expression of PD‐L1 gene could be upregulated by TNFα.^24^ We analyzed the PD‐L1 promoter region in the JASPR database, and identified a recognition site of NFKB2 at the −458 site of PD‐L1 promoter region (Figure 3C). Luciferase reporter assay further confirmed the regulation of NFKB2 on PD‐L1 promoter that NFKB2 overexpression increased PD‐L1 promoter activity and vice versa, NFKB2 knockdown significantly inhibited the promoter activity, in HEK293T and MC38 cells (Figure 3D). Considering the crucial role of PD‐L1/PD1 signaling in T‐cell exhaustion, the effects of NFKB2 on CD8^+^ T‐cell exhaustion were further evaluated. Intrahepatic infiltrating immune cells were isolated from the entire livers from spontaneous MC38 mouse models and profiled using CyTOF, and 33 immune cell subsets with specific marker genes were identified by PhenoGraph analysis of single‐cell protein expression profiles (Figure 3E). Pd‐1^+^ T cells were significantly increased in the samples from Nfkb2‐overexpressed MC38 mouse models, whereas the proportion of Cd8^+^ T and IFN‐γ cells was significantly decreased in these samples (Figure 3F). Furthermore, flow cytometry (FACS) assays of the Cd45^+^ cells from in situ transplanted tumors of both MC38 and CT26 mouse models also revealed that Nfkb2 overexpression caused a significant increased proportion of Pd‐1^+^ T cells, as well as a decreased proportion of Cd8^+^ T cells and a reduced IFN‐γ secretion (Figure 3G). We further examined the impact of NFKB2 on immune cells within the tumor microenvironment. Our findings demonstrated a significant reduction in the expression of CD4, CD8, and granzyme B (GZMB) in Nfkb2‐overexpressing MC38 tumor tissues and CRC tumor samples with high NFKB2 expression (Figure 3H). This indicates that NFKB2 suppresses T‐cell infiltration and impairs T‐cell effector functions in the tumor microenvironment. Although NFKB2 did not exhibit cancer‐promoting biological functions in the nude mouse‐based in vivo model (as mentioned in Figure S3B), it promotes T‐cell exhaustion by regulating PD‐L1 expression in CRC cells, thereby facilitating CRC progression.

### 2.4 STAT2 participates in the NFKB2‐modulated PD‐L1 expression in CRC cells

**FIGURE 4:**
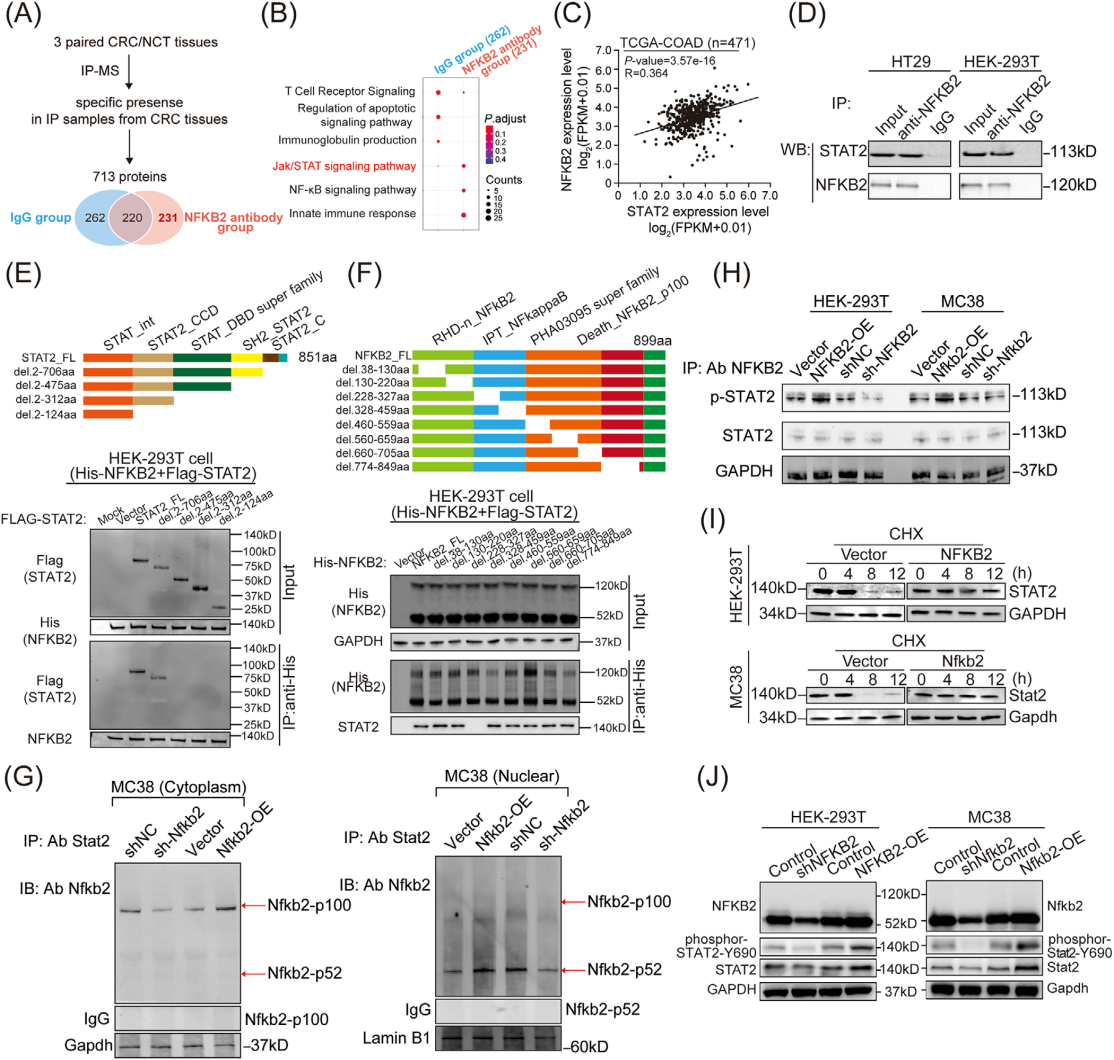
Nuclear factor kappa B subunit 2 (NFKB2) directly interacts with signal transducer and activator of transcription 2 (STAT2) in colorectal cancer (CRC). **(A)** The schematic of the identification of NFKB2‐interacting proteins in CRC tissues. **(B)** Kyoto Encyclopedia of Genes and Genomes enrichment analysis based on the proteins interacting with NFKB2 or not in CRC tissues. **(C)** The mRNA levels of NFKB2 were positively correlated with those of STAT2 in the TCGA‐COAD cohort (data source: ENCORI project). Pearson's correlation coefficient and statistical significance are indicated. **(D)** Immunoprecipitation assay confirmed the binding between NFKB2 and STAT2 in HT29 and HEK‐293T cells. **(E)** Top: schematic of STAT2 deletions. Bottom: IP of NFKB2 with Flag‐STAT2 deletions in HEK‐293T cells. FL, full‐length protein. **(F)** Top: schematic of NFKB2 deletions. Bottom: IP of NFKB2 deletion constructs (del.) with STAT2 in HEK‐293T cells. **(G)** IP experiments were conducted to assess the major forms of NFKB2 protein that interacted with STAT2 in the cytoplasm and nucleus of MC38 cells overexpressing or knocked out for Nfkb2. **(H)** IP experiments were carried out to investigate the primary forms of STAT2 protein that bound to NFKB2 in HEK‐293T and MC38 cells overexpressing or knocked out for NFKB2. **(I)** The impact of NFKB2 on the half‐life of STAT2 protein in HEK‐293T and MC38 cells. Cells were treated with 25 mM cyclohexanomycin (CHX) for the indicated times (0, 4, 8, and 12 h). **(J)** The effects of NFKB2 on the phosphorylation of STAT2 protein in HEK‐293T and MC38 cells. GAPDH served as an internal control.

To explore the molecular mechanism underlying the biological functions of NFKB2 in CRC tissues, the immunoprecipitation (IP) assay followed by mass spectrometry were performed to identify the protein partners of NFKB2 (Figure 4A). Particularly in the CRC samples, 231 proteins that interacted with NFKB2 could be enriched into Jak/STAT signaling pathway, NF‐κB signaling pathway, and innate immune response process by KEGG enrichment analysis (Figure 4B). Among these candidate proteins, STAT2, which had been proposed to play a positive role in the tumorigenesis,^25^ showed a positive correlation with NFKB2 in the mRNA levels of the TCGA‐COAD cohort (Figure 4C). Moreover, the interaction between NFKB2 and STAT2 were further confirmed in HT29 and HEK‐293T cells (Figure 4D). To investigate the interaction sites between NFKB2 and STAT2, truncation experiments based on the distinct functional domains of NFKB2 and STAT2 sequences were performed. IP assay with deletion mapping of these two proteins revealed that the SH2 domain of STAT2 (475aa‐706aa) was crucial for its interaction with NFKB2 (Figure 4E), and the N‐terminal half region of the IPT domain (228aa−327aa) of NFKB2 was essential for its binding to STAT2 (Figure 4F). Then, we isolated the cytoplasm and nucleus protein from MC38 cells and performed IP experiments, respectively, to examine the major forms of NFKB2 that bind to STAT2. The results showed that NFKB2‐p100 is the primary form of NFKB2 that binds to STAT2 in the cytoplasm, while NFKB2‐p52 is the main form that interacts with STAT2 in the nucleus (Figure 4G). Additionally, the protein primarily involved in the interaction between NFKB2 and STAT2 is the phosphorylated form of STAT2, rather than total STAT2 (Figure 4H). Furthermore, NFKB2 overexpression increased the half‐life of the STAT2 protein in HEK‐293T and MC38 cells (Figure 4I), and consequently led to changed levels of total STAT2 and Tyr690‐phosphorylated STAT2 (Figure 4J).

**FIGURE 5:**
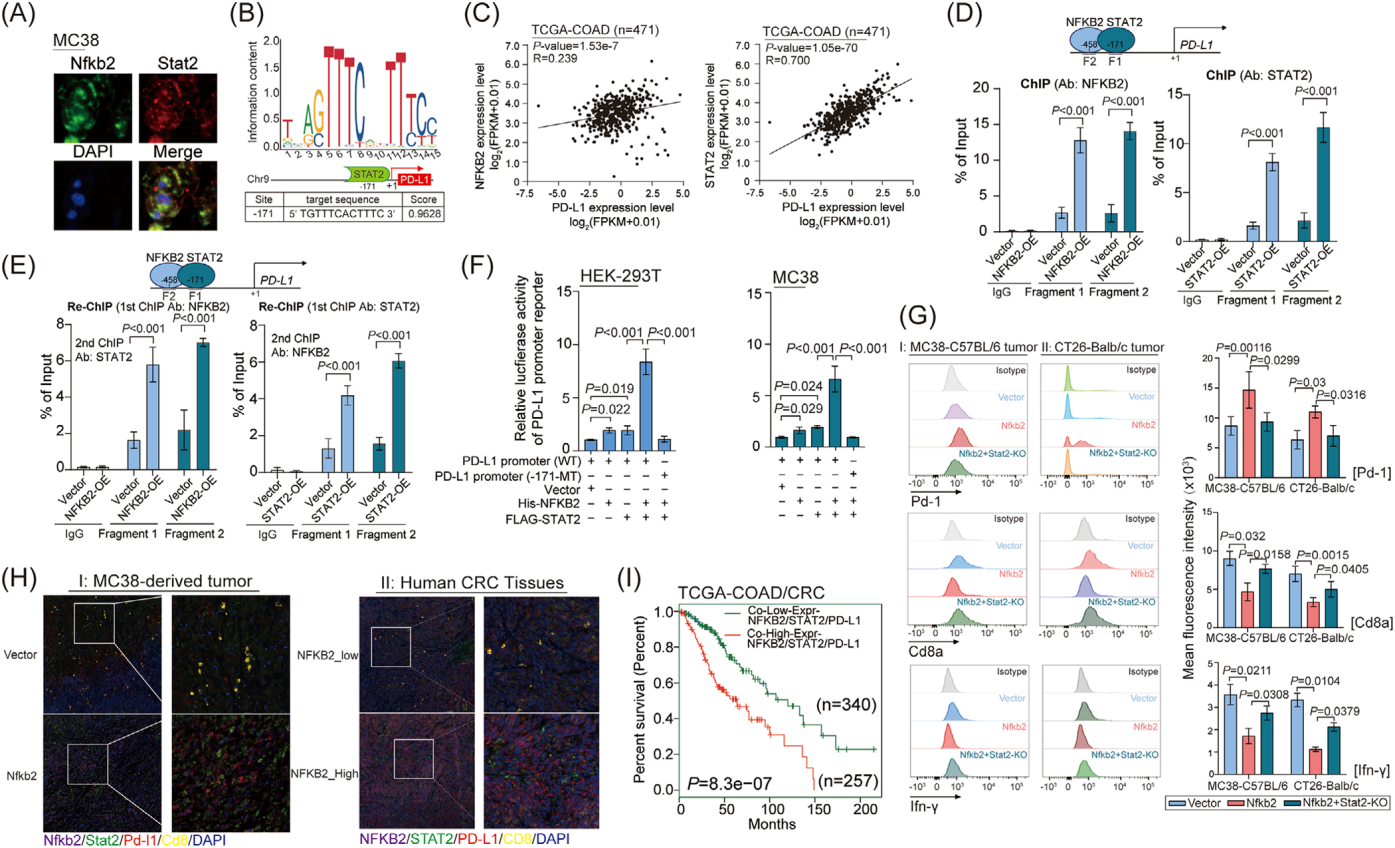
Nuclear factor kappa B subunit 2 (NFKB2) synergistically upregulates programmed death ligand 1 (PD‐L1) through interaction with signal transducer and activator of transcription 2 (STAT2). **(A)** Immunoco‐localization analysis of Nfkb2 and Stat2 in the subcutaneous xenografts derived from Nfkb2‐MC38 cells in C57BL/6 mouse model. Scale, 100 μm. **(B)** The predicted STAT2 binding site in the PD‐L1 promoter region (the JASPR database). **(C)** The mRNA levels of PD‐L1 were positively correlated with those of NFKB2 or STAT2 in the TCGA‐COAD cohort (data source: ENCORI project), respectively. Pearson's correlation coefficient and statistical significance are indicated. ChIP **(D)** and Re‐ChIP **(E)** experiments were performed to evaluate the binding of NFKB2 protein complex (or STAT2 protein complex) to the PD‐L1 gene promoter region sequences (Fragment 1 and Fragment 2) in HEK‐293T‐vector and HEK‐293T‐NFKB2‐OE (or STAT2) cells. **(F)** A luciferase reporter assay was performed to assess the impact of STAT2 and NFKB2 on the activity of the PD‐L1 promoter. **(G)** The expression levels of Pd‐1, Cd8a, and IFN‐γ in the intrahepatic Cd45^+^ cells obtained from the subcutaneous xenografts of Nfkb2‐MC38, Nfkb2/Stat2‐KO‐MC38, and Vector‐MC38 groups in C57BL/6 mouse model, or from the subcutaneous xenografts of Nfkb2‐CT26, Nfkb2/Stat2‐KO‐CT26, and Vector‐CT26 groups in Balb/c mouse model. The expression levels were examined by flow cytometry (FACS) and presented as mean fluorescence intensity (MFI). **(H)** Immunofluorescence assay was used to analyze the expression of NFKB2, STAT2, PD‐L1, and CD8 in tumor tissues of the vector‐MC38/ NFKB2‐MC38 subcutaneous xenograft mouse model and NFKB2‐low/NFKB2‐high colorectal cancer (CRC) tumor tissues. The images on the left represent a 20‐fold magnification field, while those on the right depict a 63‐fold magnification field. **(I)** The CRC patients with co‐high expression of NFKB2/STAT2/PD‐L1 represented poorer outcomes in the TCGA‐COAD cohort. The statistical significance was assessed by the log‐rank test. Our description of the position information for the regions of interest within the promoter is based on the transcription start site (TSS) being designated as 0. Values are expressed as mean ± SEM, *n* = 3 in (**D–G**).

Moreover, Nfkb2 and Stat2 co‐localized in the nucleus of MC38 cells (Figure 5A), and a highly conserved STAT2 recognition site was observed at the −171 site of PD‐L1 promoter (Figure 5B). Not surprisingly, the PD‐L1 mRNA levels correlated with the mRNA levels of NFKB2, and especially highly correlated with the mRNA levels of STAT2 in the TCGA‐COAD cohort (Figure 5C). Considering the bioinformatics prediction of potential binding sites for both NFKB2 and STAT2 within the PD‐L1 promoter region, sequences at −171 (Fragment1) and −458 (Fragment2) positions were designed to investigate the binding of STAT2 and NFKB2. ChIP experiments revealed that both NFKB2 and STAT2 complexes were able to bind at the −171 and −458 sites (Figure 5D), suggesting that NFKB2 and STAT2 may form a complex binding within the PD‐L1 promoter region. Subsequently, we performed Re‐ChIP experiments and observed that after two rounds of ChIP, effective binding was detected at both −171 and −458 sites, indicating stable complex formation of NFKB2 and STAT2 in the PD‐L1 promoter region (Figure 5E). Luciferase assays were conducted to analyze whether the binding of NFKB2 and STAT2 as a complex could synergistically enhance PD‐L1 transcriptional activity. The results demonstrated that the transfection of NFKB2 alone or STAT2 alone resulted in increased transcriptional activity of PD‐L1. However, the co‐transfection of NFKB2 and STAT2 showed a more significant enhancement of PD‐L1 transcriptional activity, suggesting a synergistic effect between NFKB2 and STAT2 in promoting PD‐L1 transcriptional activity (Figure 5F). Additionally, overexpression of STAT2 resulted in an increase in both PD‐L1 mRNA levels and protein levels and vice versa (Figure S5A,B). Therefore, these findings confirmed that NFKB2 could interact with STAT2 and result in upregulation of PD‐L1. As the amino acid sequences of the crucial regions for the interaction of these two genes are highly conserved in human and murine sources (NFKB2, https://www.ncbi.nlm.nih.gov/gene/4791/ortholog/?scope=7776; STAT2, https://www.ncbi.nlm.nih.gov/gene/6773/ortholog/?scope=32523), the evidence could reasonably be obtained on the immune cells in subcutaneous tumor‐bearing models of MC38 and CT26 tumors by FCAS analysis, which confirmed the promoting effects of Nfkb2 on the proportion of Pd‐1^+^ T cells, together with the inhibitory effects of Nfkb2 on the proportion of Cd8^+^ T cells and IFN‐γ secretion, were remarkably reversed by Stat2 knockout (Figure 5G). In further spatial localization analysis, we observed that there is an increased expression of STAT2 and PD‐L1 in tumor tissues overexpressing Nfkb2 as well as in CRC samples with high NFKB2 expression. Additionally, these samples showed decreased infiltration of CD8^+^ T cells (Figure 5H). Importantly, co‐expression of NFKB2, STAT2, and PD‐L1 was associated with poor prognoses in CRC patients in the TCGA‐COAD database (Figure 5I). These results suggested the regulatory effects of the NFKB2‐STAT2 protein complex on PD‐L1 expression and provided evidence on the contribution of this protein complex on the inflammatory and immune responses in CRC.

### 2.5 NFKB2 inhibitor Rg5 impacts the tumorigenesis of CRC cells with blocking NFKB2‐STAT2 interaction

**FIGURE 6:**
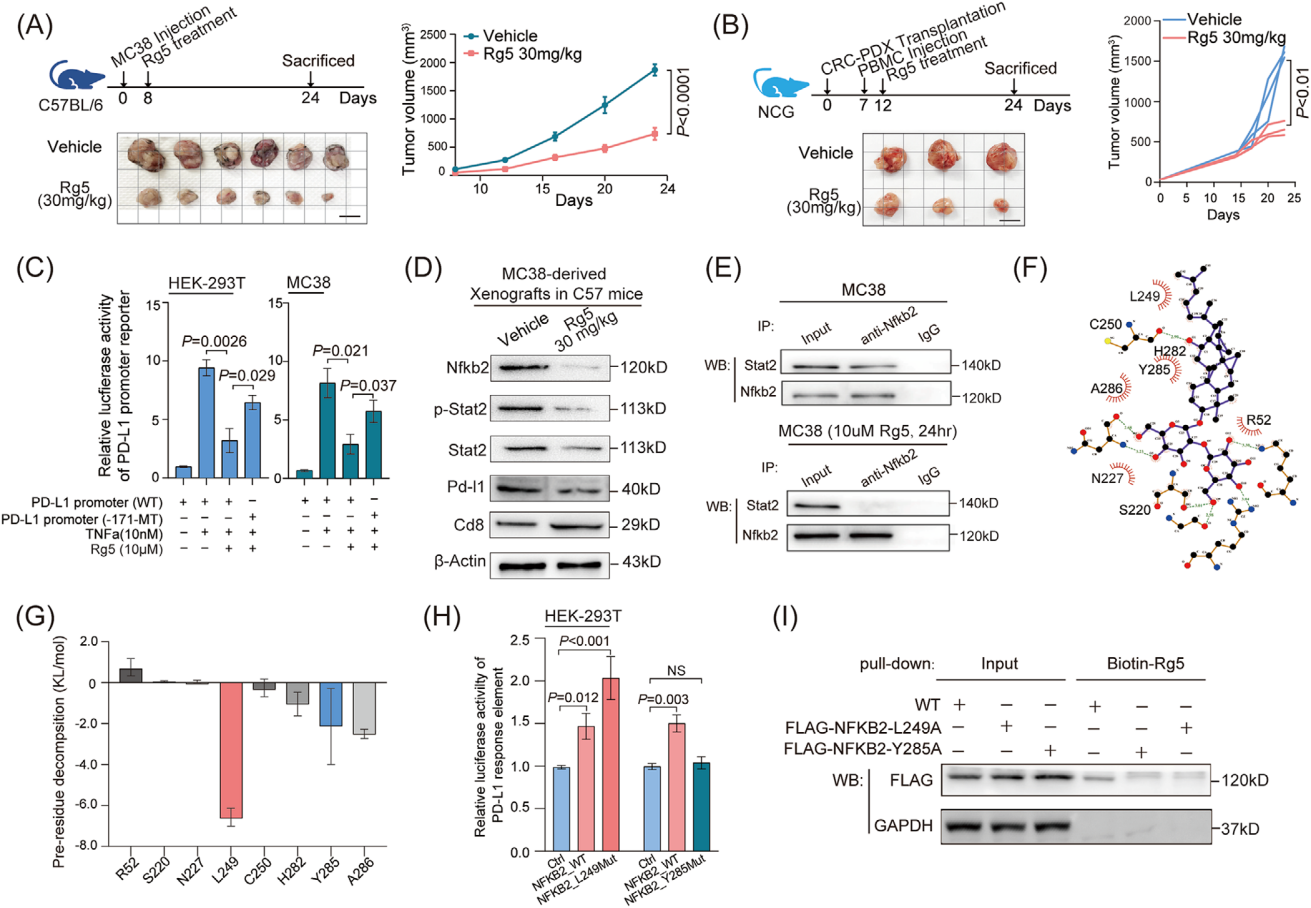
Nuclear factor kappa B subunit 2 (NFKB2) inhibitor Rg5 effectively suppresses the NFKB2/signal transducer and activator of transcription 2 (STAT2)‐induced effects in colorectal cancer (CRC). **(A)** The tumor volumes of the MC38‐derived subcutaneous xenografts treated with or without Rg5 in C57BL/6 mouse model. The standard and grid lines were 1 cm long. **(B)** The tumor volumes of the CRC‐PDX‐derived subcutaneous xenografts treated with or without Rg5 in NCG mouse model. The standard and grid lines were 1 cm long. **(C)** Rg5 inhibited NF‐κB signaling‐induced programmed death ligand 1 (PD‐L1) promoter activation, mainly relying on the existence of −171 STAT2‐recognite site in PD‐L1 promoter region. **(D)** The protein levels of Nfkb2, phosphor‐Stat2, Stat2, Pd‐l1, and Cd8a in the MC38‐derived subcutaneous xenografts treated with or without Rg5 in C57BL/6 mouse model. β‐Actin served as an internal control. **(E)** Rg5 treatment dissociated the interaction between Nfkb2 and Stat2 in MC38 cells. **(F)** Structural modeling of Rg5 in the binding pocket of NFKB2 protein. **(G)** Box plot of the per‐residue decomposition energy of eight key residues and details of the binding pose of Rg5 with the lowest binding energy. **(H)** Luciferase reporter assay was used to detect the expression of PD‐L1 after NFKB2 mutation at L249 and Y285 sites. **(I)** The mutant of L249 or Y285 of NFKB2 impacted the binding ability of Rg5 to this protein in MC38 cells. Values are expressed as mean ± SEM, *n* = 3 in (**B, C, and H**), *n* = 6 in (**A**), and *n* = 10 in (**G**).

Subsequently, specific inhibitors for NFKB2 were screened from ginsenosides (ALK, CK, PPD, Rg3, Rg5, Rb1, Rb2, Rh2, Rh4, Rk1, and CK), which were isolated and identified from ginseng,^26^ a traditional Chinese medicine with a range of pharmacological efficacy including immune‐enhancing effects. Luciferase reporter assays in a Hela‐based p52‐response‐element reporter system showed that Rg5 significantly inhibited NFKB2 activities as a transcriptional factor (Figure S6A). Also, Rg5 significantly reduced the NFKB2 protein levels in CRC cells (Figure S6B). The direct interaction between Rg5 and NFKB2 protein were proved by microscale thermophoresis (MST) assay, with a binding affinity of 5.1 μM for these two molecules (Figure S6C). In particular, the tumor volumes were both significantly lower in the Rg5‐administered MC38 tumor‐bearing mice than those in the control mice (Figure 6A). Moreover, Rg5 treatment was administered to the humanized NCG mice carrying patient‐derived xenograft (PDX) and resulted in the significant inhibition of the growth of these CRC xenografts (Figure 6B). As expected, TNFα significantly increased the transcriptional activity of the PD‐L1 promoter. However, the presence of Rg5 led to a significant suppression of this activity. Furthermore, when the mutant NFKB2 recognition site was introduced, it attenuated the inhibitory effect of Rg5 on PD‐L1 promoter activity (Figure 6C). Consistently, treatment with Rg5 led to a reduction in NFKB2 protein levels, STAT2 protein levels, STAT2 phosphorylation levels, Cd8 protein levels, and PD‐L1 protein levels (Figure 6D). Furthermore, immunoprecipitation results confirmed that Rg5 treatment significantly inhibited the binding of Nfkb2 and Stat2 within MC38 cells (Figure 6E). These findings provide evidence for the regulatory influence of Rg5 on NFKB2 in modulating the transcriptional activity of the PD‐L1 promoter. Based on the observed effects of Rg5 on Nfkb2, we hypothesized that Rg5 potentially binds to specific sites within the Nfkb2 protein. Through computational analysis, we identified that Rg5 could target the Arg52, Ser226, Leu249, Asp251, and Tyr285 sites of NFKB2 (Figure 6F). These sites mostly located in the N‐terminal half region of IPT domain (228aa−327aa) of NFKB2 were essential for its binding to STAT2. Among these predicted sites, Leu249 and Tyr285 displayed the lowest binding energies with Rg5 by GMX‐MM‐PBSA analysis (Figure 6G), suggesting greater accessibility to Rg5. Furthermore, the mutation of the L249 site on NFKB2 can increase the transcriptional activity of the PD‐L1 promoter more effectively, while the mutation of the Y285 site suppresses NFKB2‐induced enhancement of the transcriptional activity of the PD‐L1 promoter (Figure 6H). Co‐immunoprecipitation assays were performed to confirm the impact of specific mutations in NFKB2 on its binding to biotin‐labeled Rg5. The results revealed that mutations in the L249 or Y285 sites of NFKB2 significantly disrupted its association with biotin‐labeled Rg5 (Figure 6I). Cumulatively, Rg5, a small‐molecule drug targeting NFKB2, could lead to the disassociation of the NFKB2‐STAT2 complex, thus interfering with the PD‐1/PD‐L1 signaling in CRC.

### 2.6 Synergy between Rg5 administration and PD‐1 antibody treatment leads to the combination effects on CRC immunotherapy

**FIGURE 7:**
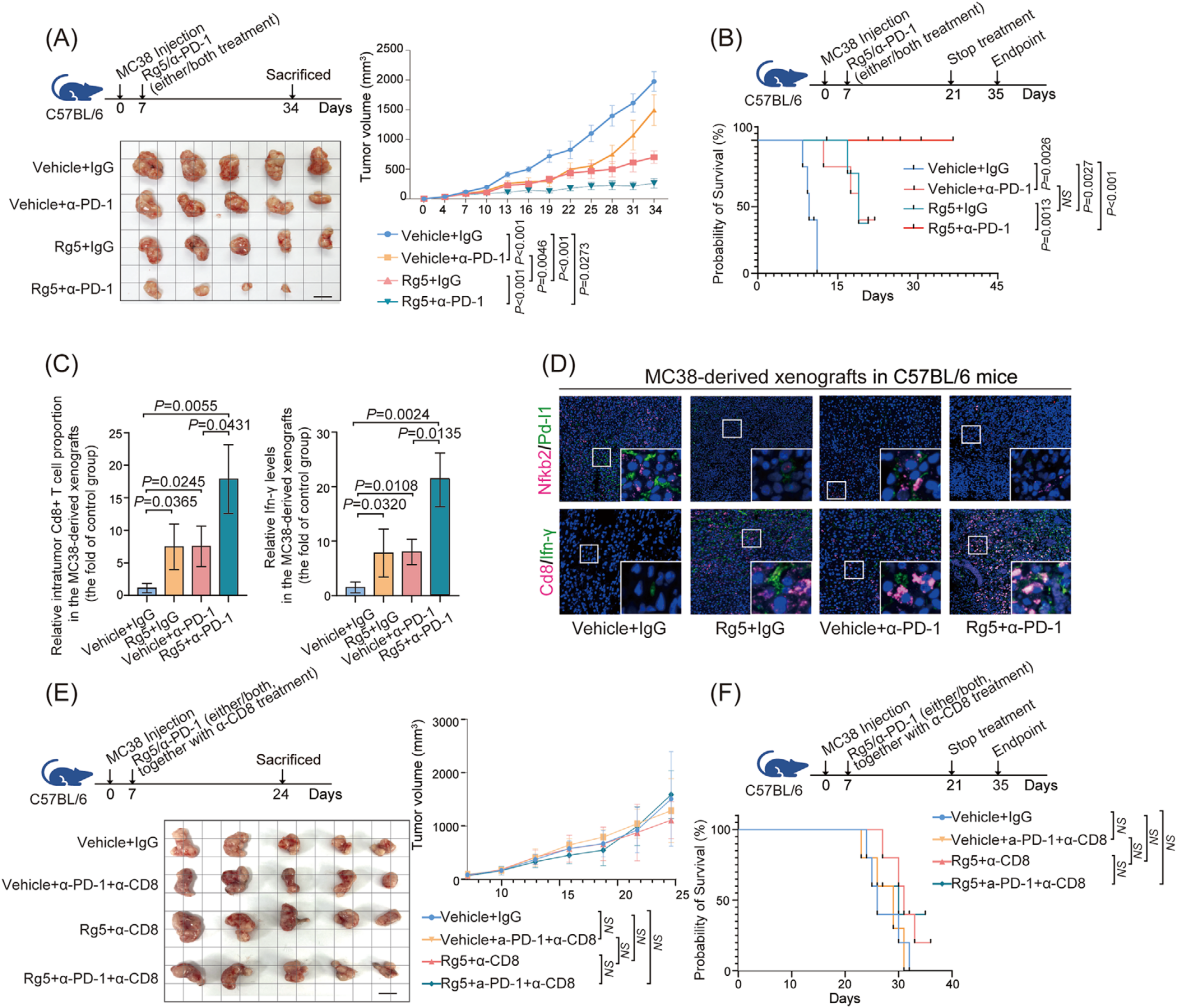
Nuclear factor kappa B subunit 2 (NFKB2) inhibitor Rg5 enhances the therapeutic effects of targeting the immune checkpoint blockade programmed death‐1 (PD‐1)/programmed death ligand 1 (PD‐L1) in the MC38‐C57BL/6 mouse model. **(A)** Tumor volumes of the MC38‐derived subcutaneous xenografts treated with vehicle control, anti‐PD‐1 mAb monotherapy (α‐PD‐1, 100 μg/mouse), Rg5 (30 mg/kg), or a combination therapy comprising both anti‐PD‐1 mAb and Rg5 in C57BL/6 mouse model. The standard and grid lines were 1 cm long. **(B)** Rg5 combined with PD‐1 antibody significantly prolonged the survival time of MC38‐C57BL/6 mouse model. **(C)** The intratumor Cd8^+^ T‐cell proportion and the expression levels of interferon γ in the MC38‐derived subcutaneous xenografts treated with indicated strategies in C57BL/6 mouse model. The expression levels were examined by enzyme linked immunosorbent assay (ELISA) and presented as the foldchange of the control group. **(D)** The expression levels of Nfkb2, Pd‐l1, Cd8, and IFN‐γ in the MC38‐derived subcutaneous xenografts treated with indicated strategies in C57BL/6 mouse model. The expression levels were examined by immunoco‐localization and the representative images are as shown. DAPI (blue) was used to visualize the nuclei. **(E)** Tumor volumes of the MC38‐derived subcutaneous xenografts treated with vehicle control, anti‐PD‐1 mAb monotherapy (α‐PD‐1, 100 μg/mouse), Rg5 (30 mg/kg), or a combination therapy comprising both anti‐PD‐1 mAb and Rg5 in C57BL/6 mouse model, under antibody‐mediated CD8^+^ T‐cell depletion (additionally supplied with anti‐CD8, 100 μg/mouse). The standard and grid lines were 1 cm long. **(F)** Anti‐CD8 supplements abolished the prolonged survival time of the group treated with Rg5 and PD‐1 antibody in MC38‐C57BL/6 mouse model. Values are expressed as mean ± SEM, *n* = 5 in (**A and E**) and *n* = 3 in (**C**).

Previous studies have reported that non‐canonical NF‐κB signaling can improve the therapeutic effects of antibodies targeting PD‐1.^27^ Given that Rg5, which directly targets NFKB2, could can significantly suppressed PD‐L1 expression in CRC cells, we further evaluated the potential synergistic impacts of Rg5 treatment on the immunotherapy for PD1/PD‐L1 checkpoint were further evaluated. Both Rg5 administration and PD‐1 antibody treatment exhibited therapeutic effects on subcutaneous MC38 tumors in syngeneic mouse model, and the combination treatment of Rg5 and PD‐1 antibody led to a more effective reduction in tumor volume than either monotherapy (Figure 7A). Moreover, the synergic therapeutic effects of Rg5 and PD‐1 antibody were strongly illustrated by the survival time of the MC38‐bearing mice (Figure 7B), which were much longer than those in either monotherapy group (32 days of median survival time for the control group, 54 days for the PD‐1 antibody monotherapy group, 45 days for the Rg5 monotherapy group, and more than 120 days for the combination treatment group). Furthermore, the proportion of intratumor Cd8^+^ T cells and the IFN‐γ levels in the xenograft samples from the combination therapy group were also significantly higher than those in the samples from the two monotherapy groups (Figure 7C). Immunofluorescence staining assays revealed that Rg5 treatment led to significant reduced protein levels of Nfkb2 and Pd‐l1, in addition to the distinctive effects of PD‐1 antibody on Pd‐1/Pd‐l1 signaling (Figure 7D, the above). Both Rg5 administration and PD‐1 antibody treatment enhanced the proportion of intratumor Cd8^+^ T cells and the IFN‐γ levels, and the combination therapy enhanced these effects (Figure 7D, the bottom). The reduced tumor volumes, together with the promoted intratumor CD8^+^ T‐cell infiltration and IFN‐γ levels, were also found in Rg5/PD‐1 antibody‐treated humanized PDX model in NOD/SCID mice (Figure S7A,B), supporting the immunotherapeutic effects of Rg5‐PD‐1 combined treatment. To further confirm the involvement of immune cells in the therapeutic effects of Rg5, the activity of Cd8^+^ T cells were nullified in MC38‐bearing mice by specific CD8 antibodies. Strikingly, no more distinct changes in tumor volume were found in tumor‐bearing mice lacking effective Cd8^+^ T cells, either in Rg5 monotherapy groups or the combination therapy group (Figure 7E), indicating that Cd8^+^ T cells are essential for the therapeutic effects of Rg5. As expected, tumor‐bearing mice depleted of Cd8^+^ T cells showed no significant difference from the control mice in median survival time (Figure 7F). Collectively, these results demonstrated that Rg5 can synergistically promote the efficacy of PD‐L1 antibodies in CRC treatment with the support of CD8^+^ T cells.

In conclusion, the findings of the present study demonstrated the frequently overexpressed NFKB2 in advanced CRC and lead to a promoting PD‐1/PD‐L1 signaling, thus promoting the immune escape and metastatic ability of CRC cells. NFKB2 interacts with STAT2, which results in an increase in phosphorylated STAT2 levels and the promotion of PD‐L1 expression. Targeting NFKB2 significantly promotes the PD‐1 antibody immune checkpoint blockade therapy, with the crucial contribution of CD8^+^ T‐cell infiltration (graphic abstract image).

## 3 DISCUSSION

This study's findings indicated a positive correlation between the upregulation of NFKB2 and poor prognosis in patients with advanced CRC with hepatic metastasis. Our data suggest that the promotion of CRC growth by NFKB2 is dependent on a competent immune system, leading us to hypothesize that the upregulation of NFKB2 is a factor in the immune evasion of CRC.

The NF‐κB and STAT signaling pathways are both crucial for antitumor immunity, and the dysregulation of these pathways frequently co‐occurs in solid cancers. IL‐6 has been implicated as a link between these pathways.^28^, ^29^, ^30^ Our studies in mouse models of gastric mucosa showed that abnormal expression of Stat1 and Stat3 mRNA was accompanied by the upregulation of Nfkb1 and activation of JAK‐STAT signaling by cytokines secreted by T and myeloid cells. These findings underscore the importance of further research on the role of NFKB2 in the immune evasion of CRC and the potential therapeutic implications of targeting this pathway. Other studies have also confirmed that Bcl‐3 promotes CRC tumorigenesis through alternative NF‐κB signaling. High levels of RelB, NFKB2 p52, and Bcl‐3 have been associated with lower survival rates17. These findings provide valuable insights into the underlying molecular mechanisms of NFKB2 in CRC and offer a promising direction for the development of new therapeutic strategies for this disease.^31^, ^32^ Thus, abnormal NF‐κB signaling can lead to abnormal JAK/STAT signaling.^31^, ^33^, ^34^

STAT2 regulates PD‐L1 expression by binding to the −171 site upstream of its promoter region. The results of the present study showed that PD‐L1 transcriptional activity was significantly increased after NFKB2 binding to STAT2 in CRC cells. The interaction between STAT2 and NFKB2 is dependent on the crucial role played by the SH2 domain of STAT2 (475aa‐706aa), with phosphorylation at Y690 being a potentially significant post‐translational modification. Other studies on gastric cancer have shown that the release of TNF‐α and IL‐6 activates NF‐κB signaling and upregulates PD‐L1 expression in tumor cells, consequently promoting the immune escape of tumor cells.^35^ The present study revealed that the proportion of intratumoral CD8^+^ T cells increased under PD‐1/PD‐L1 blockade with an anti‐PD‐1 antibody, resulting in the inhibition of CRC tumor growth. However, PD‐1 antibody‐based treatments for CRC are limited and only considered suitable in approximately 6% of cases (i.e., MSI‐H positive patients), since the majority of patients, especially those with solid tumors, show no response to anti‐PD‐1 or anti–PD‐L1 therapies.^36^, ^37^ Our observation that Rg5 combined with PD‐1 antibody can block PD‐L1/PD‐1 binding to significantly increase intratumoral CD8^+^ T‐cell populations and IFN‐γ release suggests an alternative option for therapeutic inhibition of CRC progression.

T‐cell exhaustion is often characterized by high expression of immune checkpoint genes (e.g., PD‐1, TIGIT, and CTLA‐4) and progressive loss of T‐cell function, including hierarchical loss of cytokine production (e.g., IL‐2 and IFN‐γ).^38^, ^39^, ^40^ The present study revealed that MC38 subcutaneous tumor growth was significantly reduced under NFKB2 inhibition by Rg5 in immunocompetent but not immune‐deficient mice. Several research revealed the regulation of Rg5 treatment on NF‐κB signaling pathway. Rg5 could ameliorate lung inflammation by inhibiting the phosphorylation of NF‐κB as well as the translocation of p65 into the nucleus.^41^ In animal models, Rg5 treatment has been reported to reduce cerebral infarction volume and brain neurological dysfunction of I/R rats in vivo, with a decreased p65 protein level.^42^ Rg5 also alleviates cisplatin‐induced nephrotoxicity in mice through decreasing TNFα and P65 levels, thus inhibiting the related inflammation, oxidative stress, and apoptosis.^43^ Our study reveals that Rg5 exerts anti‐cancer effects by reducing PD‐L1 expression through modulating the stability of the NFKB2/STAT2 protein complex. Additionally, immunofluorescence and enzyme linked immunosorbent assay (ELISA) experiments revealed that the presence of Rg5‐mediated suppression of NFKB2 led to an increase in the numbers of CD8^+^ T cells as well as secretion of IFN‐γ. Overall, these findings collectively indicate that NFKB2, through its interaction with STAT2, can expedite the process of CD8^+^ T‐cell exhaustion. Blocking the NF‐κB signaling pathway may be effective in the treatment of sporadic CRC. PD‐L1 gene expression is regulated by the inflammatory cytokines TNF‐α and IL‐6 secreted by infiltrated macrophages in gastric cancer.^35^ Herein, we found that NFKB2 and STAT2 could form a transcription factor complex in CRC cells and regulate the transcription of PD‐L1. NF‐κB regulation of PD‐L1 supports tissue homeostasis and promotes tissue healing under cellular stress and acute inflammation; however, it is largely dysregulated in CRC tumors.^44^ It is therefore unsurprising that only a relatively small population of MSI‐H positive CRC patients can benefit from the clinical application of PD‐1/PD‐L1 signal blockade.^45^, ^46^ Expanding the range of CRC candidates who could benefit from treatments targeting PD‐1/PD‐L1 represents an important goal in CRC therapy.^47^ A recent multicenter and multiteam study has revealed a correlation between genetic defects in the alternative pathway of human NF‐κB and the production of autoantibodies, as well as susceptibility to viral diseases such as COVID‐19 pneumonia. The presence of these autoantibodies and their potential to neutralize the activity of type I interferons has been confirmed by researchers.^48^ This finding suggests that NFKB2 serves not only as a potential target for tumor immunotherapy but also potentially plays a crucial role in viral diseases. The small molecule drug Rg5, which we have discovered, may have broader applications beyond tumor immunotherapy. In conclusion, the present study highlights the key role played by NFKB2 in the development and progression of CRC. Copy number amplification of the 10q24.32 locus in patients with advanced CRC leads to increased NFKB2 expression and its binding with STAT2, resulting in the activation of the PD‐L1/PD‐1 axis and immune escape by cancer cells. The findings of the present study suggest that NFKB2 represents a promising therapeutic target for advanced CRC. The small molecule inhibitor Rg5 effectively blocks NFKB2 binding with STAT2, resulting in decreased PD‐L1 transcription and increased immune surveillance, as evidenced by the enhanced proportion of CD8^+^ T cells in the tumor microenvironment and improved response to PD‐1 blockade immunotherapy in CRC‐bearing mice. Our study provides a basis for further exploration of the potential clinical application of NFKB2 inhibition in CRC treatment.

## 4 MATERIALS AND METHODS

### 4.1 Cell lines and mice

The CRC cell lines MC38, CT26, and HT‐29 were purchased from the American Type Culture Collection (ATCC) and cultured according to the manufacturer's instructions. These cells were characterized using short tandem repeat markers by Genewiz, Inc. and were confirmed to be mycoplasma free (last tested in 2017). Note that 5‐ to 6‐week‐old BALB/c nude, C57BL/6, BALB/c, NOD/SCID, and NOD‐Prkdc^em26Cd52^IL2^rgγem26Cd52^/Gpt (NCG) mice were purchased from Gempharmatech and were housed under pathogen‐free conditions, in temperature‐controlled holding rooms (20−22°C), with 30%−34% humidity, and on a 12 h on (6 a.m.) and off light cycle (6 p.m.).

### 4.2 Human patient sample collections

We collected 79 paired human CRC tissues and NCTs at the Affiliated Hospital of Jiangnan University, and 86 paired CRC tissues and NCTs at Fudan University Zhongshan Hospital. These samples were the same as those used in our previous study.^49^ In this study, we utilized different sample cohorts. Cohort 1 (Table S1 *n* = 70) consisted of DNA samples collected from paired human CRC tissues and corresponding non‐cancerous tissues (corresponding to Figure 1D,E). Cohort 2 (*n* = 50) comprised RNA samples collected from paired human CRC tissues and corresponding non‐cancerous tissues (corresponding to Figure 1F). Cohort 3 (*n* = 21) included RNA samples collected from CRC patients with liver metastasis (corresponding to Figure 1G,H). All patient materials were obtained with informed consent, and this study was conducted with the permission of the Clinical Research Ethics Committees.

### 4.3 CRC xenograft tumor model

For in vivo tumor formation assays, 2 × 10^6^ HT‐29 cells were suspended in 200 μL serum‐free DMEM and subcutaneously injected into the flank of each nude mouse (female BALB/c nude, five per group). Tumor sizes were measured twice a week as soon as the tumors were measurable, and tumor volumes were calculated using the following formula: *V* (mm^3^) = width^2^ (mm^2^) × length (mm)/2.

### 4.4 CRC syngeneic mouse model and hepatic metastasis model

For the in vivo tumor formation assays, 5 × 10^5^ MC38 cells were suspended in 200 μL serum‐free DMEM medium and subcutaneously injected into the flank of each C57BL/6 mouse (female , five per group). Note that 1 × 10^6^ CT26 cells were suspended in 200 μL serum‐free DMEM medium and subcutaneously injected into the flank of each BALB/c mouse (female BALB/c, five per group). In Rg5 separate treatment assay, Rg5 was administered by gavage once daily at a dose of 30 mg/kg from the seventh day on.

For Rg5 and PD‐1 antibody combination therapy, 7 days later, Rg5 was administered by gavage once daily at a dose of 30 mg/kg, and 100 μg/mouse anti‐PD‐1 (pembrolizumab, BE0273, BioX cell) was injected into the abdomen every 2 days. To deplete mCd8^+^ T cells in C57BL/6 mice, 200 μg/mouse anti‐Cd8 depletion mAb (BE0117, BioX cell) was administrated intraperitoneally to C57BL/6 mouse 1 day before pembrolizumab treatment, followed by weekly intraperitoneal injections of anti‐CD8 mAb for 25 days. IgG2b mAb (BE0086, BioX cell) was injected intraperitoneally into control C57BL/6 mice on the same days as the control.

In the orthotopic tumor model, female BALB/c or C57BL/6 mice aged 6−8 weeks were initially subcutaneously injected with either 1 × 10^6^ CT26‐luciferase cells or 5 × 10^5^ MC38‐luciferase cells into the right flank. Upon reaching a tumor size of approximately 500 mm^3^, the mice were euthanized, and the tumors were excised, cut into 1–2 mm fragments, and collected in cold PBS after removal of necrotic areas. Anesthesia was administered to the mice before surgery, and a tumor fragment was implanted onto the serosal layer of the cecum through a laparotomy procedure. Closure of the muscle and skin was performed using 5‐0 absorbable suture and 5‐0 monofilament nylon suture, respectively. The mice were administered an intraperitoneal injection of d‐luciferin (2.5 mg/100 μL in PBS) for in vivo bioluminescent imaging using an IVIS system (PerkinElmer).

### 4.5 Humanized PDX NCG mouse models

Female NCG mice (5−6 weeks old) received myeloablation using busulfan (30 mg/kg, i.p.; B2635, Sigma‐Aldrich) at the day before CRC‐PDX transplantation. The injection of PBMCs (5 × 10^6^ cells/mouse, i.v. Schbio, PBMNC050C) was administrated on day 7. The engraftment levels of hCD45^+^ cells were determined 2 weeks post‐PBMCs transplantation by flow cytometric quantification of peripheral blood hCD45^+^ cells. For humanized PDX (Hu‐PDX) tumor experiments, Rg5 treatments were started when implanted PDX tumors reached a volume of 100–200 mm^3^. The control group received intraperitoneal injections of PBS. Rg5 was administered once daily at a dose of 30 mg/kg, and 100ug anti‐PD‐1 was injected into the abdomen every 2 days.

### 4.6 Statistical analysis

All results are presented as mean values ± SEM. The Student's *t*‐test or one‐way analysis of variance was performed to evaluate the differences between two groups or more than two groups, respectively, followed by Dunnett's multiple comparisons tests. Wilcoxon tests were used to analyze Mrna levels in paired human samples, and Mann–Whitney tests were used to analyze Mrna levels in grouped human samples. The Kaplan–Meier method and log‐rank test were used to determine the differences in survival rates between the NF‐κB signaling pathway in the low‐ and high‐expression groups. All statistical analyses were performed using R v3.5.1. *p*‐Values <  0.05 were considered statistically significant.

Detailed Materials and Methods are available in the Supplementary Information.

## AUTHOR CONTRIBUTIONS

J.Z., L.Y., Z.W., and X.W. designed and supervised this project; F.M., Z.L., Y.L., F.Y., and E.W. performed most of the experiments, and J.Z., L.Y., Z.W., and X.W. analyzed the results; M.S. carried out bioinformatics analyses; X.S. provided patients samples and clinical and pathological information; J.Z., LY., Z.L., Y.L., Z.W., and X.W. contributed the conception of schematic representation to figures; J.Z., L.Y., Z.W., and X.W. wrote the paper with comments from all authors. All authors have read and approved the final manuscript.

## CONFLICT OF INTEREST STATEMENT

The authors declare no conflicts of interest.

## ETHICS STATEMENT

The human research was approved by the Medical Ethics Committee, Affiliated Hospital of Jiangnan University (approval date: Jan 21, 2022). The animal research was approved by the Institutional Animal Care and Use Committee of Shanghai Immunocan Biotechology Co., Ltd. (approval number: YMNK‐IACUC‐F006).

## Supporting information

Supporting Information

## Data Availability

All datasets involved in this study can be viewed in the Gene Expression Omnibus (https://www.ncbi.nlm.nih.gov/geo/query/acc.cgi?acc=GSE250065) or data availability part of the corresponding articles. All data pertinent to this study, whether generated or analyzed, are comprehensively presented in this manuscript and its Supporting Information. For any additional inquiries or requests, interested parties are encouraged to contact the corresponding authors.
