# Supplementary material for: NFKB2 mediates colorectal cancer cell immune escape and metastasis in a STAT2/PD‐L1‐dependent manner
Source: MedComm (2020). 2024 Apr 24;5(5):e521. [Article in Catalan] doi: 10.1002/mco2.521 (PMC11042535; doi:10.1002/mco2.521)
Supplement: Supplementary file 1 — Supporting Information [file MCO2-5-e521-s001.docx]

**NFKB2 mediates colorectal cancer cell immune escape and metastasis in a STAT2/PD-L1-dependent manner**

Jiwei Zhang^1*^, Fen Ma^1^, Zhe Li^2^, Yuan Li^1^, Xun Sun^3^, Mingxu Song^4^, Fan Yang^1^, Enjiang Wu^1^, Xiaohui Wei^1*^, Zhengtao Wang^1*^, Li Yang^1*^

^1^Shanghai Key Laboratory of Compound Chinese Medicines, The MOE Key Laboratory for Standardization of Chinese Medicines, Institute of Chinese Materia Medica, Shanghai University of Traditional Chinese Medicine, Shanghai 201203, China.

^2^Academy of Integrative Medicine, Shanghai University of Traditional Chinese Medicine, Shanghai, 201203, China.

^3^Gastrointestinal surgery, Longhua Hospital, Shanghai University of Traditional Chinese Medicine, Shanghai 200032, China.

^4^Human Reproductive and Genetic Center, Affiliated Hospital of Jiangnan University, Wuxi, Jiangsu 214062, China.

Jiwei Zhang, Fen Ma, Zhe Li and Yuan Li contributed equally to this work.

**Running title:** NFKB2 promotes immune escape of CRC.

^*^**Corresponding authors:**

**Xiaohui Wei**, The MOE Key Laboratory for Standardization of Chinese Medicines, Institute of Chinese Materia Medica, Shanghai University of Traditional Chinese Medicine, Shanghai, China. Email: xhweixh@163.com

**Zhengtao Wang**, The MOE Key Laboratory for Standardization of Chinese Medicines, Institute of Chinese Materia Medica, Shanghai University of Traditional Chinese Medicine, Shanghai, China. Email: [ztwang@shutcm.edu.cn](mailto:ztwang@shutcm.edu.cn)

**Li Yang**, The MOE Key Laboratory for Standardization of Chinese Medicines, Institute of Chinese Materia Medica, Shanghai University of Traditional Chinese Medicine, Shanghai, China. Email: [yl7@shutcm.edu.cn](mailto:yl7@shutcm.edu.cn)

**Jiwei Zhang,** The MOE Key Laboratory for Standardization of Chinese Medicines, Institute of Chinese Materia Medica, Shanghai University of Traditional Chinese Medicine, Shanghai, China. Email: joezhang@shutcm.edu.cn.

**SUPPLEMENTARY MATERIALS AND METHODS**

**Ginsenoside Rg5**

Ginsenoside Rg5 (Purity: 95%~99%) was purchased from Chengdu Biopy Phytochemicals Co., Ltd. (Chengdu, China), Catalogue No.: BP1651 and Cas No.: 186763-78-0.

**Function analysis and Binding site prediction**

The clusterProfiler R package (version 4.0.5) was used for functional enrichment of different gene sets. The GSVA R package (version 1.40.1) from Bioconductor was used to assign pathway activity (c2BroadSets), which were described in the molecular signature database^1^. Gene Set Enrichment Analysis (GSEA) in the clusterProfiler R package (version 4.0.5) to evaluate activation of hallmark pathways from the molecular signature database. The binding site of transcription factor and target gene promoter region was predicted by the JASPR database (https://jaspar.genereg.net/).

**Bulk RNAseq library preparation**

Total RNA was extracted for the RNA sample preparations. Poly-T oligo-attached magnetic beads were used for purifying mRNA. Random hexamer primer and M-MuLV Reverse Transcriptase (RNase H-) were used for synthesizing first strand cDNA. DNA Polymerase I and RNase H were used for Second strand cDNA synthesis. cDNA fragments with 370~420 bp in length were selected. TruSeq PE Cluster Kit v3-cBot-HS (Illumia) was used for clustering of index-coded samples. Illumina Novaseq 6000 platform was used for sequencing and 150 bp paired-end reads were generated.

**Differential gene analysis**

Raw sequence reads were trimmed for adptor squence/low-quality sequence using fastqc (0.11.5). Trimmed sequence reads were mapped GRCm38/mm10 using STAR (2.7.9). Read count extraction and quantification were performed using featureCounts (2.0.1). Differential expression analysis between two groups was used the DESeq2 R package (1.32.0). Functional enrichment analysis was used Metascape (https://metascape.org/gp/index.html).

**Lentivirus construction and infection**

The primer sequences targeting human NFKB2 (Gene ID: 4791, NCBI Reference Sequence: NM_001077494.3) and STAT2 (Gene ID: 6773, NCBI Reference Sequence: NM_001385110.1), as well as mouse Nfkb2 (Gene ID: 18034, NCBI Reference Sequence: NM_001177369.1) and Stat2 (Gene ID: 20847, NCBI Reference Sequence: NM_019963.2), were utilized for the amplification of genomic RNA from normal tissues. Subsequently, the amplified sequences were cloned into the pWPXL lentiviral vector to generate Case9. The viral particles were harvested 48 h after HEK 293T cells were transfected with the generated plasmid, packaging plasmid psPAX2, and VSV-G envelope plasmid pMD2.G using Lipofectamine 2000 reagent (Invitrogen, USA). The recombinant lentiviral transduction units were used to infect MC38 and HT-29 cells (Sigma-Aldrich, USA) in the presence of 1 μg/ml polyethylene. Information regarding all the primers used is provided in **Table S1**.

**Luciferase reporter assay**

To generate the reporter gene plasmid, the nucleotide sequences encoding human NFKB2 (or its murine homolog, Nfkb2) or STAT2 (or its murine homolog, Stat2) are integrated upstream of the luciferase reporter gene. The human or mouse plasmid containing the PD-L1 promoter is separately co-transfected with the reporter gene plasmid into HEK-293T or MC38 cells. After 48 hours, the transfected cells are harvested. Cell lysis is performed using a lysis buffer, followed by the addition of the luciferase assay reagent. The luminescent signal intensity is measured using a luminometer to quantify reporter gene activity.

**Co-IP assay and Mass Spectrometry (MS)** **analysis**

Cell lysates were prepared using RIPA lysis buffer containing a cocktail protease inhibitor. After incubating MC38 cells at 4°C for 2 hours, the lysate was centrifuged at 13000 rpm at 4°C for 15 minutes. The resulting supernatant was transferred to a new tube and centrifuged again at 13000 rpm at 4°C for 5 minutes. The upper layer was collected and stored at 4°C. The protein concentration in the extract was determined using a BCA kit.

For IP lysis, the cell lysates were centrifuged at 12,000 rpm for 15 minutes at 4°C, and the supernatants were collected. After preclearing the lysates using Protein A+G agarose beads, anti-NFKB2 or control IgG was added, followed by incubation with Protein A+G agarose beads overnight at 4°C. The lysates were then subjected to MS analysis to identify proteins specifically bound to NFKB2. Detailed information about the antibodies used is provided in **Table S2**.

**Chromatin Immunoprecipitation (ChIP) and Re-ChIP Assays**

Cells were crosslinked with 1% formaldehyde at a concentration of 1×10^6^ cells per ml for 5 minutes at room temperature with rotation. The crosslinking reaction was stopped by adding 0.125M glycine for 10 minutes. The resulting pellet was lysed using 1 mL of lysis buffer, and after centrifugation, the pellet was washed once with digestion buffer and then incubated with MNase enzyme at 37 °C for 20 minutes. The digestion was stopped by adding 0.5M EDTA. The mixture was sonicated, and the pellet was discarded after centrifugation. For immunoprecipitation, the sheared chromatin was incubated overnight at 4 °C with specific antibodies, Protein G beads, and dilution buffer. The eluted protein-DNA complexes were purified using a DNA purification kit. Furthermore, a Re-ChIP assay was performed using the eluted chromatin from the first ChIP to undergo a second round of immunoprecipitation with the indicated antibodies.

**Protein truncation test**

RNA was extracted from human-derived cells to obtain NFKB2 and STAT2 transcripts. Reverse transcription and PCR techniques were used to synthesize cDNA encoding NFKB2 and STAT2, with various lengths based on their functional domains for subsequent protein truncation experiments. The resulting cDNA fragments were separated and purified using agarose gel electrophoresis. His and FLAG tags were introduced at the C-terminus of the NFKB2 and STAT2 fragments through PCR amplification with specific primers. The tagged NFKB2 and STAT2 sequences were then ligated onto plasmid vectors. The constructed fusion protein expression plasmids were verified by sequencing to confirm the accuracy and consistency of the sequences. Subsequently, these validated plasmids were separately transfected into HEK-293T cells. Following 72 hours of transient transfection, the cells were harvested for Co-IP experiments.

**Microscale thermophoresis**

The recombinant protein NFKB2 was diluted to a concentration of 200nM with PBS+0.05% Tween-80 protein buffer. After incubation with dye, the mixture was harvested and transferred to PCR tubes along with Rg5 and buffer samples. The samples were then analyzed using a Monolith NT.115 instrument, and data analysis was performed using MO. Analysis software. The affinity Kd was calculated using the thermophoresis curve fitting method.

**Immunoblotting and immunofluorescence assays**

MC38 Cells were lysed in 8 mol/L urea lysis buffer with protease and phosphatase inhibitors for 30 minutes at 4°C. The lysates were cleared by centrifugation, and the proteins were separated using gel electrophoresis. After blocking the membranes, they were incubated with primary antibodies diluted in a specified solution at 4°C overnight. Subsequently, the membranes were washed and incubated with horseradish peroxidase secondary antibody. The bound antibody was detected using enhanced chemiluminescence. Immunofluorescence staining was also performed to detect the subcellular localization of Nfkb2, Pd-l1, Ifn-γ, and Cd8. After fixation and blocking, the cells were incubated with primary antibodies followed by incubation with conjugated secondary antibodies. The samples were mounted and analyzed under a fluorescence microscope. Positive samples were determined based on signal intensity, and the percentage of positive cells was calculated. Information regarding all the antibodies used is provided in **Table S2**.

**Measurement of IFNγ-secreting CD8^+^ T cells by the ELISA assay**

In the antigen-specific CD8^+^ T cell functional assay in the MC38 mouse model, 25 days after tumor inoculation, the antigen was diluted to 20 µg/mL in PBS and transferred to the first row of micropores of the PVC microtitration plate for coating. After incubation and washing, blocking buffer was added to each well to block non-specific protein-binding sites. Lymphocytes were then re-stimulated with SIINFEKEL or MC38 tumor cells for 36 hours. An anti-IFNγ antibody was pre-coated in a 96-well HTS-IP plate, and after co-culture, biotinylated anti-IFNγ antibody and avidin-horseradish peroxidase were added successively. IFN-γ spots were developed and analyzed according to the manufacturer’s instructions. Information regarding all the antibodies used is provided in **Table S2**.

**Flow Cytometry and Mass Cytometry by Time-Of-Flight (CyTOF)**

**Cell Isolation:** Tumor-infiltrating lymphocytes (TILs) were isolated from freshly excised tumor tissues. The tissues were mechanically disrupted and then digested in a mixture of 0.3 mg/ml DNase I (Sigma-Aldrich) and 0.25 mg/ml Liberase TL (Roche) at 37°C for 30 minutes. After digestion, the tissues were passed through a 40μm cell strainer to obtain single cells, which were then washed and suspended in Hank's Balanced Salt Solution (HBSS) containing 1% FBS for subsequent staining and flow cytometric analysis. For cytokine detection, the cells were treated with Cell Activation Cocktail (Biolegend, 423303) at 37°C for 6 hours.

**Antibody Staining:** The isolated cells underwent cell surface antibody staining by adding a diluted antibody mixture and incubating at 4°C for 30 minutes. Following washing, the cells were fixed with a fixation solution at 4°C for 30 minutes. Subsequently, intracellular antibody staining was performed by adding a diluted antibody mixture in permeabilization buffer (eBioscience) and incubating on ice for 45 minutes. After staining, the cells were washed and resuspended for flow cytometry and CyTOF (Fluidigm, Helios) analysis. Data analysis was conducted using FlowJo software.

**Hematoxylin and Eosin Staining (HE staining) and Multiplexed IHC staining**

Tumor tissue retrieved from the MC38 injection was subjected to fixation and paraffin embedding, and then sliced using a microtome. The sections cut from paraffin blocks were baked at 60°C for 1 h, deparaffinized, rehydrated with serial passage through xylene and graded alcohol, and finally washed in water. Hematoxylin (Sigma, H3136) solution was used for nuclear staining, and the sections were then differentiated in acid alcohol or tap water to remove excess stain. Subsequently, the sections were counterstained with eosin (Sigma, E4009) solution to visualize cytoplasmic components. Finally, the stained sections were scanned using a high-resolution digital pathology slide scanner (KFBIO, KF-PRO-120) to capture images at various magnifications.

Multiplexed immunofluorescence analysis was performed according to the manufacturer’s instructions (PerkinElmer). Stained slides were counterstained with 4’, 6-diamidino-2-phenylindole (DAPI), and the cover slipped for review. Positive samples were defined as those that contained ≥5% stained cells. Information regarding all the antibodies used is provided in **Table S2**.

**Western blot analysis**

Proteins were separated by sodium dodecyl sulfate-polyacrylamide gel electrophoresis (SDS-PAGE) and transferred to a nitrocellulose membrane (Bio-Rad, Hercules, CA, USA). The membranes were blocked with 5% non-fat milk in PBS-T and incubated with the indicated primary antibodies for 12 h. After washing five times with PBS-T, membranes were incubated with secondary antibodies for 1 h at room temperature. Information regarding all the antibodies used is provided in **Table S2**.

**SUPPLEMENTARY FIGURES AND FIGURES LEGENDS**

**Supporting Figure 1**

**
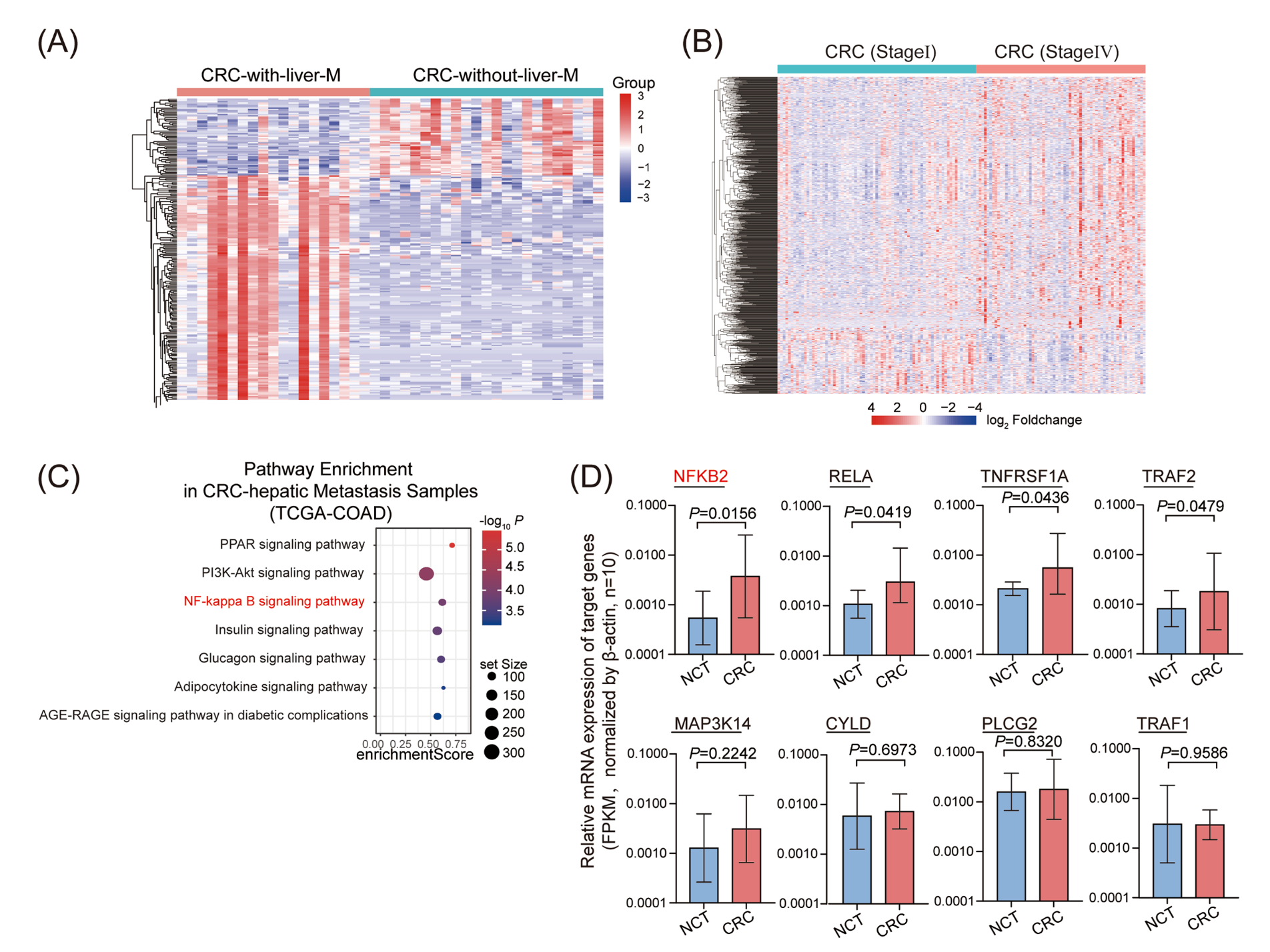
**

**Figure S1. NFKB2 was significantly upregulated in advanced CRC samples with hepatic metastasis.**

**(A)** Samples from the public database GSE81558 were analyzed for differentially expressed genes (DEGs) in CRC samples with or without hepatic metastasis. **(B)** Samples from the TCGA-COAD cohort were analyzed for DEGs in CRC samples in Stage 1 (n=75) or Stage 4 (n=54). |log2 (fold change)|> 1 and adjusted P value < 0.05 were the cutoff for screening. **(C)** Gene Ontology (GO) pathway enrichment analysis based on the DEG pattern of the TCGA-COAD cohort. **(D)** A primary validation of the mRNA levels of NF-κB related genes in an independent cohort of CRC samples. Values are expressed as mean ± SEM, n=10.

**Supporting Figure 2**

**
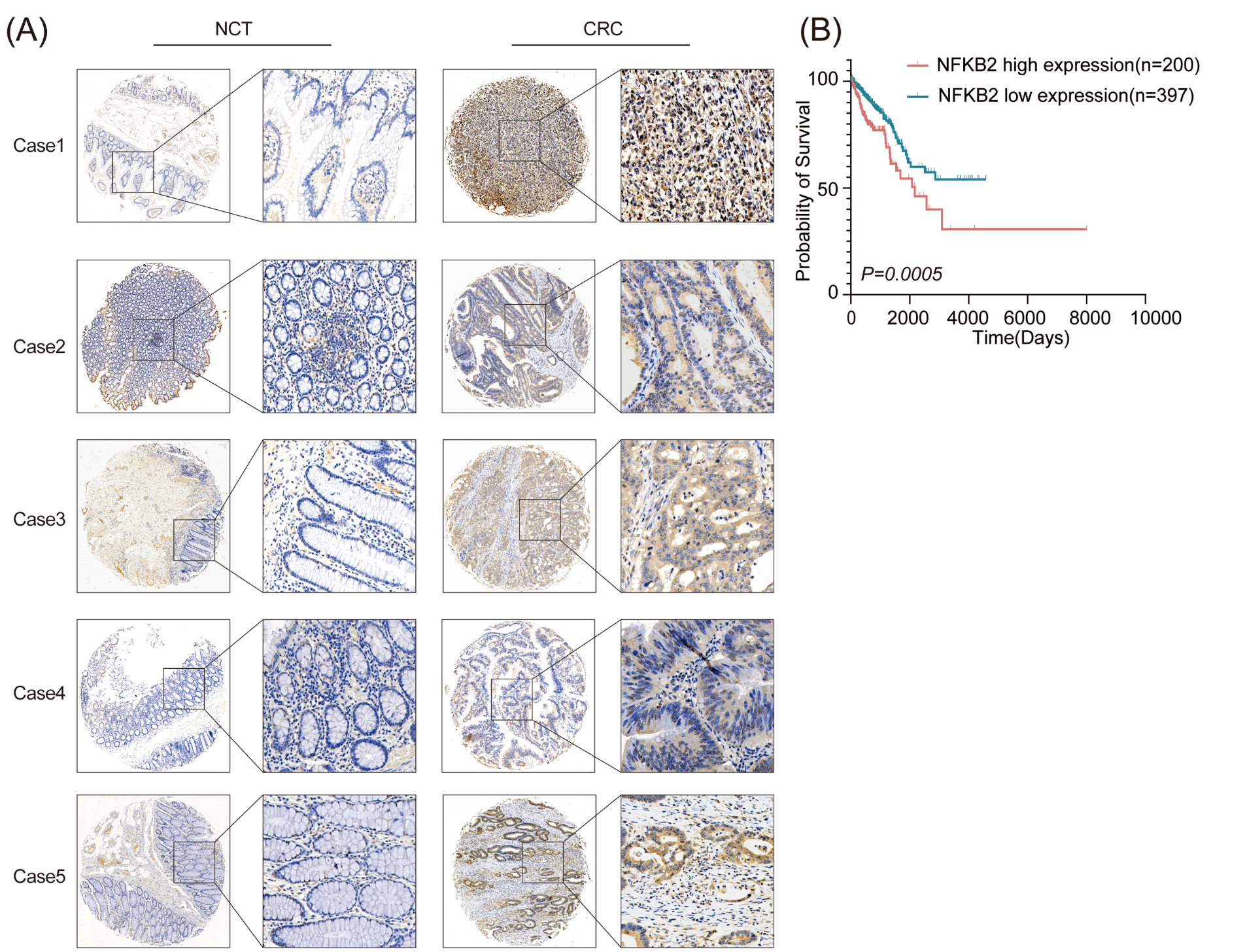
**

**Figure S2. Expression of NFKB2 in CRC patients and its correlation with prognosis.**

**(A)** Immunohistochemical analysis was performed to evaluate the expression of NFKB2 in non-cancerous tissues (NCTs) and tumor tissues of CRC patients. **(B)** Kaplan-Meier analysis of the association between mRNA levels of NFKB2 and clinical outcome in the CRC patients in TCGA-COAD cohort. Patients were stratified by the median value of NFKB2 mRNA levels. The statistical significance was assessed by the log-rank test.

**Supporting Figure 3**

**
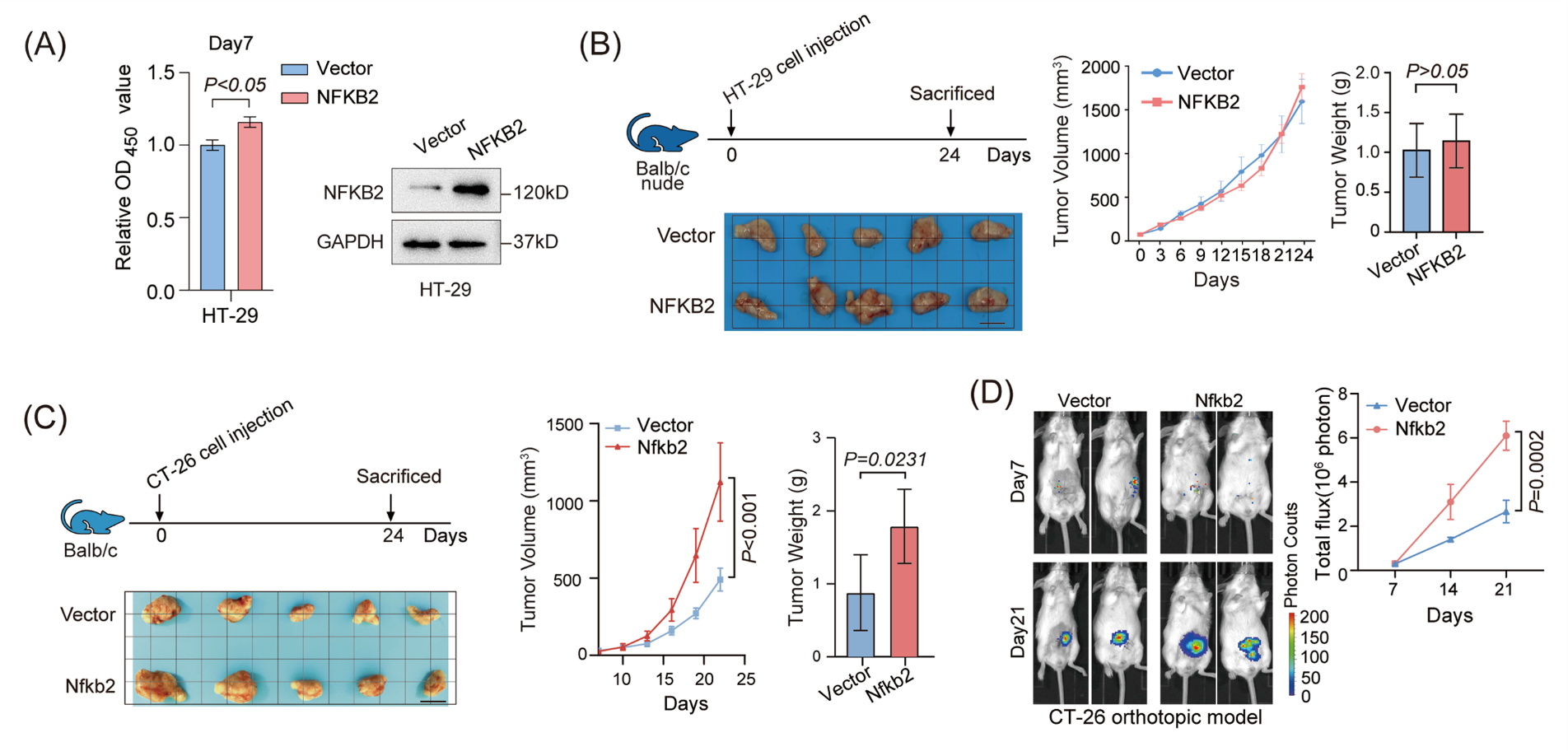
**

**Figure S3. Assessment of the biological role of NFKB2 in the HT-29 cell line and various mouse models.**

**(A) Left:** The effect of overexpressed NFKB2 on the proliferative capacity of HT29 cell lines was evaluated using the CCK8 assay. **Right:** The overexpression efficiency of NFKB2 in HT-29 cells was assessed in the WB experiment. **(B)** The tumor volumes and tumor weights of the NFKB2-HT29 and Vector-HT29 groups in Balb/c nude mouse model. The standard and grid lines were 1cm long. **(C)** The tumor volumes and tumor weights of the Nfkb2-CT26 and Vector-CT26 groups in Balb/c mouse model. The standard and grid lines were 1cm long. **(D)** Following in situ inoculation of Nfkb2-CT26-luciferase and Vector-CT26-luciferase in the Balb/c mouse intestines, the bioluminescent intensity of mice was measured at different time points. Representative bioluminescent images on day 7 and day 21 are shown on the left, accompanied by the corresponding statistical results on the right. Values are expressed as mean ± SEM, n=3 in (**A**), n=5 in (**B-D**).

**Supporting Figure 4**

**
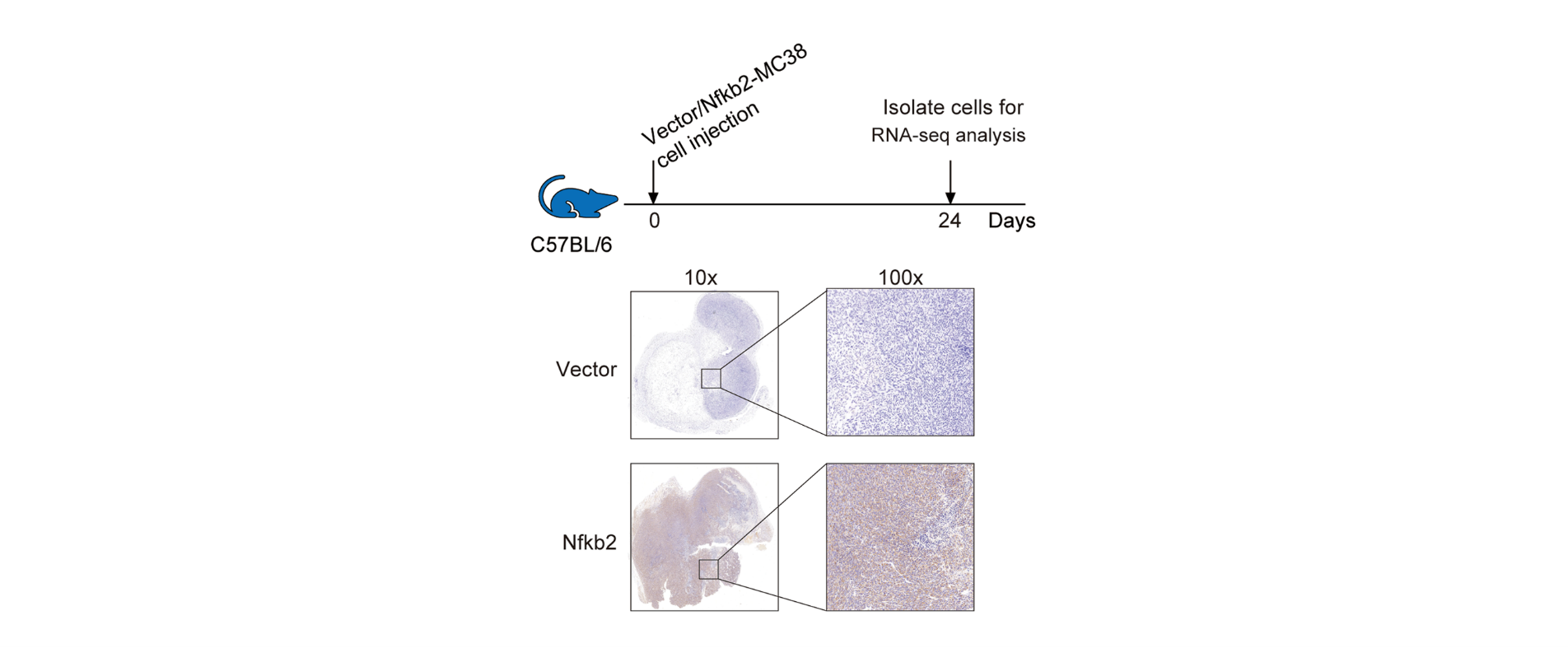
**

**Figure S4. Xenografts for RNA-sequencing assays were from the *in vivo* assays constructed with indicated MC38-derived cells in C57BL/6 mouse, and the up-regulation of Nfkb2 was verified by IHC.** Original magnification was as shown.

**Supporting Figure 5**

**
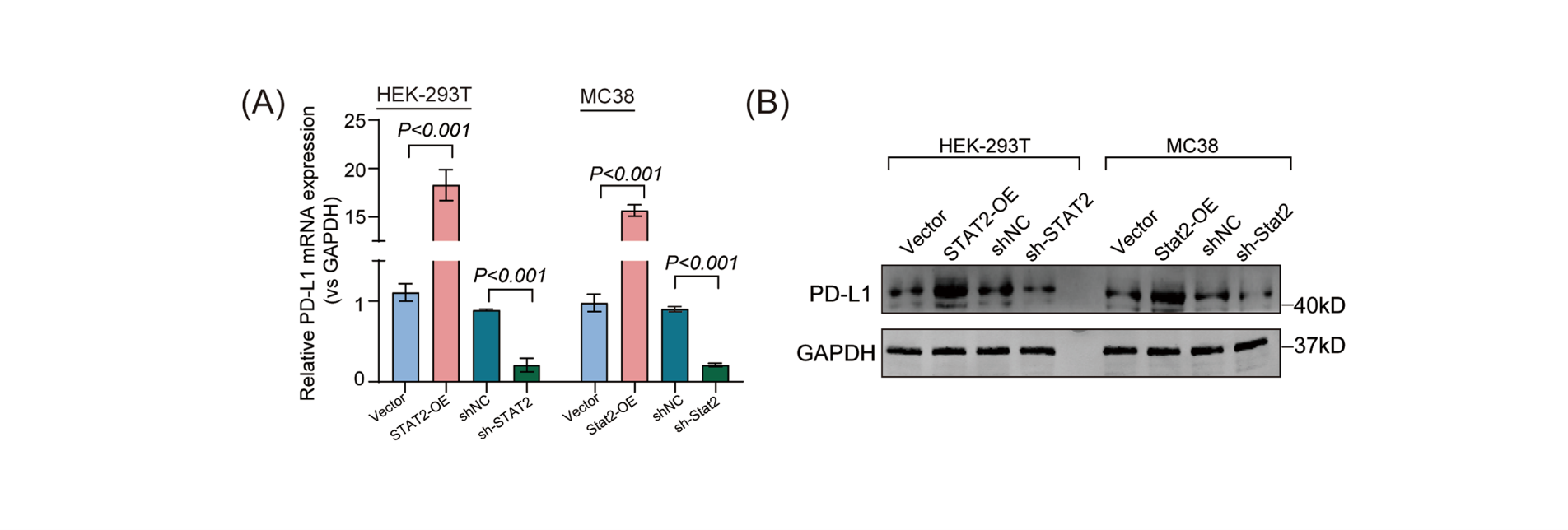
**

**Figure S5. *In vitro* experiments examining the impact of STAT2 overexpression or knockout on PD-L1 expression.**

**(A-B)** qPCR **(A)** and Western blot **(B)** were performed to assess the expression of PD-L1 in HEK-293T and MC38 cells following STAT2 overexpression or knockout. Values are presented as mean ± SEM, n=3 in (**A**).

**Supporting Figure 6**

**
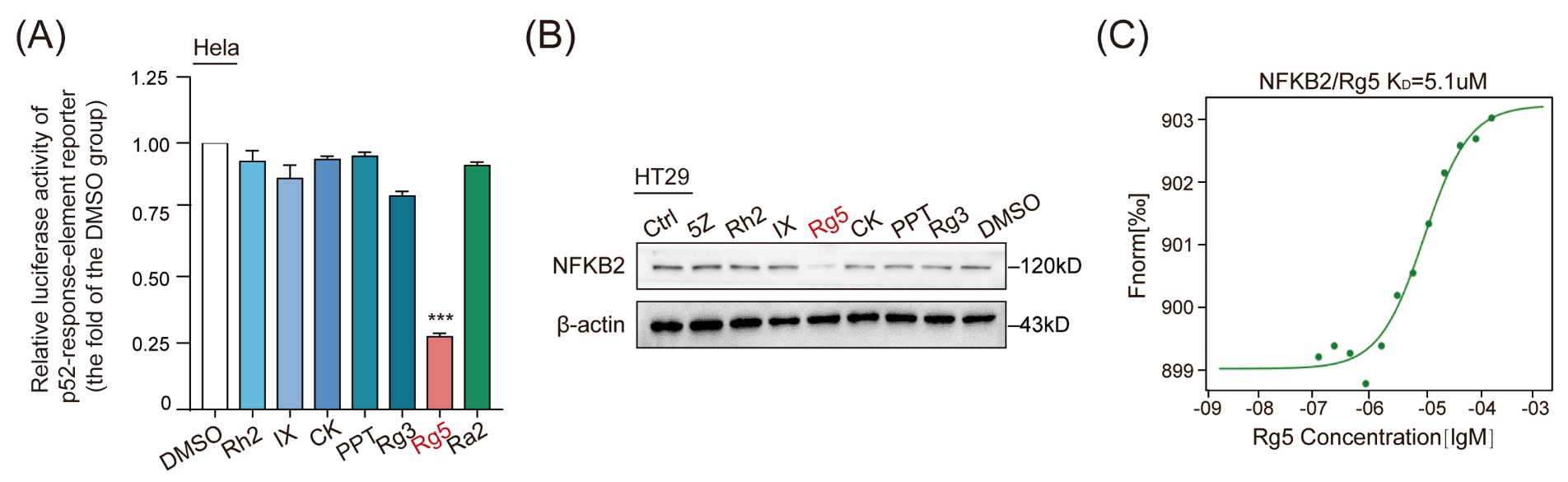
 Figure S6. Ginsenoside Rg5 is a NFKB2 inhibitor.**

**(A)** The inhibitory effects of Rg5 treatment on the luciferase activity of HeLa cells containing p52-response-element containing reporter. 10μM Ginsenosides were added to the cells, which had been transiently transfected with reporter plasmids for overnight, respectively. 24hrs later, the luciferase activities were examined. Values are expressed as mean ± SEM, n=3. **(B)** The protein levels of NFKB2 in the HT-29 cell which were treated with indicated Ginsenosides with a concentration of 10μM for 48hr. β-actin was used as internal control. **(C)** Microscale thermophoresis (MST) binding assay to determine the Kd values for the binding of Rg5 with NFKB2 (NFKB2 Kd = 5.1μM). ****P*<0.001

**Supporting Figure 7**

**
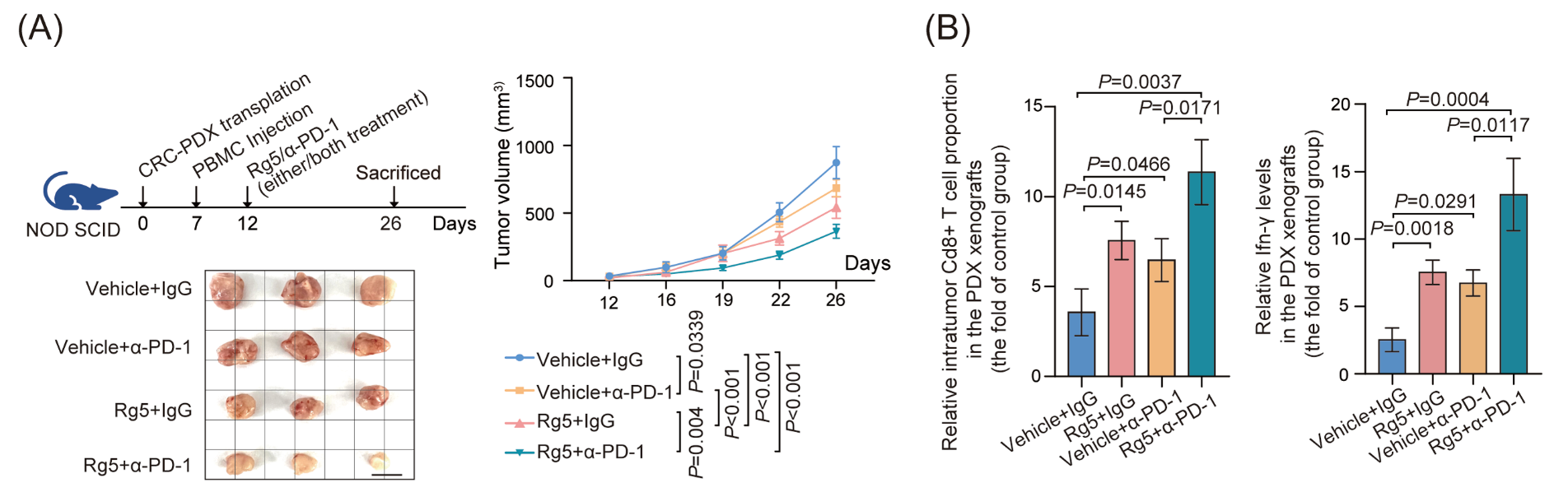
 Figure S7. NFKB2 inhibitor Rg5 enhances the therapeutic effects of targeting the immune checkpoint blockade PD-1/PD-L1 in the PDX-NOD/SCID mouse model.**

**(A)** The tumor volumes of the CRC-PDX-derived subcutaneous xenografts treated with vehicle control, anti-PD-1 mAb monotherapy (α-PD-1, 100μg/mouse), Rg5 (30mg/kg), or a combination therapy comprising both anti-PD-1 mAb and Rg5 in NOD/SCID mouse model. The standard and grid lines were 1cm long. **(B)** The intratumor Cd8^+^ T cell proportion and the expression levels of Ifn-γ in the CRC-PDX-derived subcutaneous xenografts treated with indicated strategies in NOD/SCID mouse model. The expression levels were examined by ELISA and presented as the foldchange of the control group. Values are expressed as mean ± SEM, n=3 in (**A and B**).

**Supporting Figure 8**

**Figure S8. The structure of Rg5.**

**SUPPORTING TABLES**

**Table S1.** **Primers and Antibodies for western blot, IHC, and treatment information in this study**

| Identifier | Sequence (5’ to 3’) |
| --- | --- |
| *For CRISPR-cas9 system* |  |
| Human NFKB2-SgA | TCTGCGAGCATACAGGTGTA AGG |
| Human NFKB2-SgB | AGTTTGGTCGACCGTGCAAG GGG |
| Human NFKB2-SgC | GAGAGCATCTGCGAGCATAC AGG |
| Human NFKB2-SgD | TGCTGCGAAACGTTAAGTGC AGG |
| *For real time PCR assay* |  |
| mouse Nfkb2-S1 | ATCCGGTGGAGGTCGAGATC CGG |
| mouse Nfkb2-S2 | CAGTCCCTGAGCGAGAATAG AGG |
| mouse Nfkb2-S3 | CGGGCGTGGCGGGTAAGATC CGG |
| mouse Nfkb2-S4 | TCCCTGAGCGAGAATAGAGG AGG |
| PD-L1 fragment1 Forward primer | AATTTGAAACTTCTCATCTTT |
| PD-L1 fragment1 Reverse primer | AACTGAAATAGTCTTCTAATA |
| PD-L1 fragment2 Forward primer | AAGGATGGCACCTGAAGGAGG |
| PD-L1 fragment2 Reverse primer | TTTCAGAGCAAGGAGAATCTGA |
| *For plasmids construction* |  |
| STAT2 shRNA target sequence 1 | TGTCTTCTGCTTCCGATATAA |
| STAT2 shRNA target sequence 2 | ATCAGCTTCACGGTCAAATAT |
| Stat2 shRNA target sequence 1 | TTGGCTGAGATGATCTTTAAT |
| Stat2 shRNA target sequence 2 | GGCCAGAGACAGGGCTTAATT |
| Stat2-FL-Primer 1 | ATGGCGCAGTGGGAGATGTTG |
| Stat2-FL-Primer 2 | GCTCCTAGTCCTCAGAAGGTATCAAGAGT |
| STAT2-FL-Primer 1 | ATGGCGCAGTGGGAAATGC |
| STAT2-FL-Primer 2 | GATTACAAGGATGACGACGATAA GGAAGTCAGAAGGCATCAAGGGTCC |
| STAT2-706-Primer 1 | GCGCAGTGGGAAATGCTG |
| STAT2-706-Primer 1 FLAG | GATTACAAGGATGACGACGATAA TTGCAGTTCATCCACCTGTCTATTAGA |
| STAT2-475-Primer 1 | GCGCAGTGGGAAATGCTGC |
| STAT2-475-Primer 1 FLAG | GATTACAAGGATGACGACGATAA GCTGAGCAAATTGAACCAGAGAACTG |
| STAT2-312-Primer 1 | GCGCAGTGGGAAATGCTGC |
| STAT2-312-Primer 1 FLAG | GATTACAAGGATGACGACGATAA GTGGAGCAGACGCTGTAGCA |
| STAT2-124-Primer 1 | GCGCAGTGGGAAATGCTGC |
| STAT2-124-Primer 1 FLAG | GATTACAAGGATGACGACGATAA GGCCCTCTGAGCCTGGATC |
| NFKB2 siRNA 21nt guide 1 | UCAUCAUAUUCAAUAAUACCA |
| NFKB2 siRNA 21nt passenger 1 | GUAUUAUUGAAUAUGAUGAUU |
| NFKB2 siRNA 21nt guide 2 | AUUUGAAAUCAUCAUAUUCAA |
| NFKB2 siRNA 21nt passenger 2 | GAAUAUGAUGAUUUCAAAUUG |
| Nfkb2 siRNA 21nt guide 1 | UAGCAAUUGUCCAUAUCUCUG |
| Nfkb2 siRNA 21nt passenger 1 | GAGAUAUGGACAAUUGCUACG |
| Nfkb2 siRNA 21nt guide 2 | UCAAAAUCAUCAUAUUCGGGG |
| Nfkb2 siRNA 21nt passenger 2 | CCGAAUAUGAUGAUUUUGAAU |
| Nfkb2-FL Forward primer | ATGGACAATTGCTACGATCC |
| Nfkb2-FL Reverse primer | TCAGTGCACCTGAGGCTG |
| NFKB2-FL Forward primer | ATGGAGAGTTGCTACAACCCAGGT |
| NFKB2-FL Reverse primer | CATCATCACCATCACCACGTGCACCTGAGGCTGGGG |
| NFKB2-del.38-130aa Fragment 1 Forward primer | ATGGAGAGTTGCTACAACCCAGG |
| NFKB2-del.38-130aa Fragment 1 Reverse primer | AGGGGCCATCAGCTGTTTCTG |
| NFKB2-del.38-130aa Fragment 2 Forward primer | GCCCAATTTAACAACCTGGGTGTC |
| NFKB2-del.38-130aa Fragment 2 Reverse primer | GTCAGTGCACCTGAGGCTGG |
| NFKB2-del.38-130aa Fragment 3 Forward primer | ATGGAGAGTTGCTACAACCCAGG |
| NFKB2-del.38-130aa Fragment 3 Reverse primer | GTCAGTGCACCTGAGGCTGG |
| NFKB2-del.130-220aa Fragment 1 Forward primer | ATGGAGAGTTGCTACAACCCAGG |
| NFKB2-del.130-220aa Fragment 1 Reverse primer | CCCCGGAGATTTCATGTCCTTGGGCCCCAC |
| NFKB2-del.130-220aa Fragment 2 Forward primer | CCAAGGACATGAAATCTCCGGGGGCATCAAAC |
| NFKB2-del.130-220aa Fragment 2 Reverse primer | CATCATCACCATCACCACGTGC |
| NFKB2-del.130-220aa Fragment 3 Forward primer | ATGGAGAGTTGCTACAACCCAGG |
| NFKB2-del.130-220aa Fragment 3 Reverse primer | CATCATCACCATCACCACGTGC |
| NFKB2-del.228-277aa Fragment 1 Forward primer | ATGGAGAGTTGCTACAACCCAGG |
| NFKB2-del.228-277aa Fragment 1 Reverse primer | ACATCTGTGGGGTTTGATGCCCCCGGAGATT |
| NFKB2-del.228-277aa Fragment 2 Forward primer | GGGCATCAAACCCCACAGATGTGCATAAACAGTATGC |
| NFKB2-del.228-277aa Fragment 2 Reverse primer | CATCATCACCATCACCACGTGC |
| NFKB2-del.228-277aa Fragment 3 Forward primer | ATGGAGAGTTGCTACAACCCAGG |
| NFKB2-del.228-277aa Fragment 3 Reverse primer | CATCATCACCATCACCACGTGC |
| NFKB2-del.228-327aa Fragment 1 Forward primer | ATGGAGAGTTGCTACAACCCAGG |
| NFKB2-del.228-327aa Fragment 1 Reverse primer | TCTTCCACCAGGTTTGATGCCCCCGGAGATT |
| NFKB2-del.228-327aa Fragment 2 Forward primer | GGGCATCAAACCTGGTGGAAGACAAGGAAGAGGT |
| NFKB2-del.228-327aa Fragment 2 Reverse primer | CATCATCACCATCACCACGTGC |
| NFKB2-del.228-327aa Fragment 3 Forward primer | ATGGAGAGTTGCTACAACCCAGG |
| NFKB2-del.228-327aa Fragment 3 Reverse primer | CATCATCACCATCACCACGTGC |
| NFKB2-del.460-559aa Fragment 1 Forward primer | ATGGAGAGTTGCTACAACCCAGG |
| NFKB2-del.460-559aa Fragment 1 Reverse primer | TGAGTCTCCTCGGGCGCTGCGC |
| NFKB2-del.460-559aa Fragment 2 Forward primer | AGCGCCCGAGGAGACTCAGCCATGCATCTG |
| NFKB2-del.460-559aa Fragment 2 Reverse primer | CATCATCACCATCACCACGTGC |
| NFKB2-del.460-559aa Fragment 3 Forward primer | ATGGAGAGTTGCTACAACCCAGG |
| NFKB2-del.460-559aa Fragment 3 Reverse primer | CATCATCACCATCACCACGTGC |
| NFKB2-del.560-659aa Fragment 1 Forward primer | ATGGAGAGTTGCTACAACCCAGG |
| NFKB2-del.560-659aa Fragment 1 Reverse primer | GCGTTCACGTTATGCCGATCCAGCAGAGC |
| NFKB2-del.560-659aa Fragment 2 Forward primer | GGATCGGCATAACGTGAACGCTCGCACC |
| NFKB2-del.560-659aa Fragment 2 Reverse primer | CATCATCACCATCACCACGTGC |
| NFKB2-del.560-659aa Fragment 3 Forward primer | ATGGAGAGTTGCTACAACCCAGG |
| NFKB2-del.560-659aa Fragment 3 Reverse primer | CATCATCACCATCACCACGTGC |
| NFKB2-del.660-705aa Fragment 1 Forward primer | ATGGAGAGTTGCTACAACCCAGG |
| NFKB2-del.660-705aa Fragment 1 Reverse primer | GGTGAAGGGGCCCGGAGCTTGGTG |
| NFKB2-del.660-705aa Fragment 2 Forward primer | TCCGGGCCCCTTCACCCCCTACCTCTGA |
| NFKB2-del.660-705aa Fragment 2 Reverse primer | CATCATCACCATCACCACGTGC |
| NFKB2-del.660-705aa Fragment 3 Forward primer | ATGGAGAGTTGCTACAACCCAGG |
| NFKB2-del.660-705aa Fragment 3 Reverse primer | CATCATCACCATCACCACGTGC |
| NFKB2-del.774-849aa Fragment 1 Forward primer | ATGGAGAGTTGCTACAACCCAGG |
| NFKB2-del.774-849aa Fragment 1 Reverse primer | CTCGGGTTTCTGGTGTATCACCAAGTGACAGTCCCGG |
| NFKB2-del.774-849aa Fragment 2 Forward primer | CTTGGTGATACACCAGAAACCCGAGACAAGCT |
| NFKB2-del.774-849aa Fragment 2 Reverse primer | CATCATCACCATCACCACGTGC |
| NFKB2-del.774-849aa Fragment 3 Forward primer | ATGGAGAGTTGCTACAACCCAGG |
| NFKB2-del.774-849aa Fragment 3 Reverse primer | CATCATCACCATCACCACGTGC |

**Table S2. Antibodies for western blot, IHC, flow cytometry and treatment used in this study**

| Identifier | Company | Catalog No. |
| --- | --- | --- |
| NFKB2 | PTG | 10409-2-AP |
| PD-1 | immunoway | immunoway |
| GAPDH | abcam | Ab9485 |
| CD8 | Cell Signaling Technology | 98941 |
| Pdcd1 | Cell Signaling Technology | 84651 |
| NFKB2 | abcam | Ab175192 |
| P65 | Cell Signaling Technology | 8242 |
| p-P65(s536) | santa cruz | sc-136548 |
| PD-L1 | PTG | 66248-1-Ig |
| CD8 | Cell Signaling Technology | 85336 |
| IFN-γ | U-CyTech | CT310 |
| PD-1 | BioXcell | BE0273 |
| CD8 | BioXcell | BE0117 |
| IgG2b | BioXcell | BE0086 |
| IFN-γ | abcam | Ab64029 |
| CD8 | abcam | ab238264 |
| PD-1 | BioXcell | SIM0003 |
| STAT2 | Proteintech | 16674-1-AP |
| STAT2 | Cell Signaling Technology | 72604 |
| Stat2 | Cell Signaling Technology | 4597 |
| Phospho-Stat2 (Tyr690) | Cell Signaling Technology | 88410 |
| NFKB1 | R&D Systems | AF2697-SP |
| ReIB | Santa Cruz Biotechnology | sc-166416 |
| FLAG | Cell Signaling Technology | 14793 |
| HIS | Cell Signaling Technology | 12698 |

**REFERENCE**

1 Subramanian A, Tamayo P, Mootha V K *, et al.* Gene set enrichment analysis: a knowledge-based approach for interpreting genome-wide expression profiles. *Proc Natl Acad Sci U S A.* 2005;102(43):15545-15550.
